# Selective Hydrogenation and Hydrodeoxygenation of Aromatic Ketones to Cyclohexane Derivatives Using a Rh@SILP Catalyst

**DOI:** 10.1002/anie.201916385

**Published:** 2020-05-27

**Authors:** Gilles Moos, Meike Emondts, Alexis Bordet, Walter Leitner

**Affiliations:** ^1^ Max Planck Institute for Chemical Energy Conversion 45470 Mülheim an der Ruhr Germany; ^2^ DWI-Leibniz Institute for Interactive Materials Forckenbeckstrasse 50 52056 Aachen Germany; ^3^ Institut für Technische und Makromolekulare Chemie RWTH Aachen University Worringerweg 2 52074 Aachen Germany

**Keywords:** hydrodeoxygenation, ionic liquids, nanoparticles, rhodium, supported ionic liquid phases

## Abstract

Rhodium nanoparticles immobilized on an acid‐free triphenylphosphonium‐based supported ionic liquid phase (Rh@SILP(Ph_3_‐P‐NTf_2_)) enabled the selective hydrogenation and hydrodeoxygenation of aromatic ketones. The flexible molecular approach used to assemble the individual catalyst components (SiO_2_, ionic liquid, nanoparticles) led to outstanding catalytic properties. In particular, intimate contact between the nanoparticles and the phosphonium ionic liquid is required for the deoxygenation reactivity. The Rh@SILP(Ph_3_‐P‐NTf_2_) catalyst was active for the hydrodeoxygenation of benzylic ketones under mild conditions, and the product distribution for non‐benzylic ketones was controlled with high selectivity between the hydrogenated (alcohol) and hydrodeoxygenated (alkane) products by adjusting the reaction temperature. The versatile Rh@SILP(Ph_3_‐P‐NTf_2_) catalyst opens the way to the production of a wide range of high‐value cyclohexane derivatives by the hydrogenation and/or hydrodeoxygenation of Friedel–Crafts acylation products and lignin‐derived aromatic ketones.

## Graphical abstract

**Some like it hot**: Rhodium nanoparticles immobilized on an acid‐free triphenylphosphonium‐based supported ionic liquid phase (Rh@SILP(Ph_3_‐P‐NTf_2_)) enabled ready access to high‐value cyclohexane derivatives from aromatic ketones. The product distribution could be switched with high selectivity between the completely hydrogenated (alcohol) and hydrodeoxygenated (alkane) products by changing the reaction temperature (see scheme).

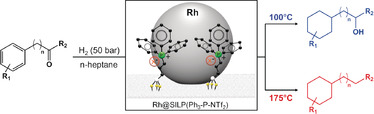

## Introduction

**Figure 1:**
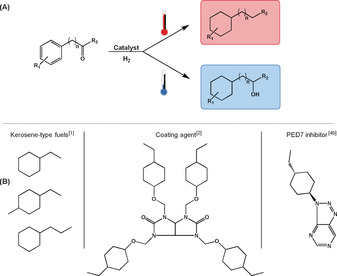
A) Pathways for the synthesis of cyclohexane derivatives through the temperature‐controlled hydrogenation or hydrodeoxygenation of aromatic ketones. B) Examples of applications of cyclohexane derivatives.

The synthesis of alkyl cyclohexane derivatives has attracted considerable attention in the past decade owing to the importance of these compounds in the transportation sector^1^ (kerosene‐type fuels) and as building blocks for the production of coating agents,^2^ liquid crystals,^3^ and pharmaceuticals.^4^ The traditional method for the synthesis of alkyl cyclohexanes consists of the hydrogenation of alkyl aromatic compounds^5^ commonly produced through Friedel–Crafts alkylation reactions. However, this pathway suffers from the limited substrate scope and often low selectivity of Friedel–Crafts alkylation (overalkylation, carbocation rearrangements).^6^ In this context, the hydro(deoxy)genation of aromatic ketones obtained, for example, through Friedel–Crafts acylation^7^ or the oxidative depolymerization of lignin^8^ appears an attractive alternative. Furthermore, the use of aromatic ketones as substrates gives the opportunity to access two classes of compounds (alkyl cyclohexanes and hydroxy‐containing cyclohexane derivatives) and thus broadens significantly the range of possible products and potential applications (Figure 1). Despite recent efforts,^9^ the development of versatile catalytic systems able to effectively hydrogenate and/or hydrodeoxygenate a large range of aromatic ketones remains a major challenge and constitutes the focus of this study.

Whereas in heterogeneous catalysis, hydrogenation reactions are mainly performed in the presence of transition‐metal nanoparticles (Ni,^10^ Ru,^11^ Rh,^12^ Pt,^13^ etc.), the subsequent hydrodeoxygenation typically requires the presence of both a metal and a strong Brønsted or Lewis acidic catalyst.^14^ Metal nanoparticles immobilized on supported ionic liquid phases (SILPs) were demonstrated to open a molecular approach to multifunctional catalytic systems with tailor‐made reactivity.^15^, ^16^ SILPs are suitable matrices for nanoparticle synthesis and stabilization. The ionic liquid structure can be readily functionalized to bring different types of actives sites in intimate contact with the active metal. Recent studies demonstrated the synthesis of monometallic16a, 16b, 16c and bimetallic15f, 16d nanoparticles on imidazolium‐based SILPs to produce catalytic systems with excellent catalytic properties for hydrogenation and hydrodeoxygenation reactions. In particular, the choice of the acid, and close proximity between the metal and acid sites were shown to be key factors in the development of effective hydrodeoxygenation catalysts.16b, 16c, 16d

## Results and Discussion

We report herein the synthesis of Rh nanoparticles immobilized on a triphenylphosphonium‐based SILP. Using the resulting Rh@SILP(Ph_3_‐P‐NTf_2_) catalyst (NTf_2_=bis(trifluoromethane)sulfonimide), aromatic ketones were effectively hydrogenated and hydrodeoxygenated without the need for an additional acid functionality. Whereas benzylic ketones were readily hydrodeoxygenated under mild conditions, non‐benzylic ketones could be selectively hydrogenated or hydrodeoxygenated depending on the temperature applied, thus providing flexible access to a wide range of substituted cyclohexanes.

The functionalized support material SILP(Ph_3_‐P‐NTf_2_) was synthesized through the condensation of a triethoxysilane‐functionalized phosphonium ionic liquid, ([triphenyl(3‐(triethoxysilyl)propyl)phosphonium]NTf_2_), on dehydroxylated silica following a modified established procedure.^17^ Analysis by DRIFT spectroscopy (see Figures S1–S3 in the Supporting Information) showed signals at 1440, 1484, 1590 cm^−1^ and 2897, 2930, 2977, 3072 cm^−1^ characteristic of the triphenylphosphine moiety. ^29^Si solid‐state NMR spectroscopy (see Figure S4) showed the presence of two types of Si species: 1) tetrafunctionalized (Q) Si with signals at −110 (Q4=Si(OSi)_4_) and −102 ppm (Q3=Si(OSi)_3_OH); and 2) trifunctionalized (T) Si with signals at −61 (T2= R‐Si(OSi)_2_OR′) and −53 ppm (T1=R‐Si(OSi)(OR′)_2_). The T2 and T1 signals correspond to the Si atoms of IL bound to the SiO_2_ surface and thus substantiate the covalent attachment of the IL on the silica support. ^1^H, ^13^C, ^31^P, and ^19^F solid‐state NMR spectroscopy further confirmed the presence of the desired triphenylphosphonium–NTf_2_ ionic liquid in the SILP(Ph_3_‐P‐NTf_2_) (see Figures S5–S8 A).

**Figure 2:**
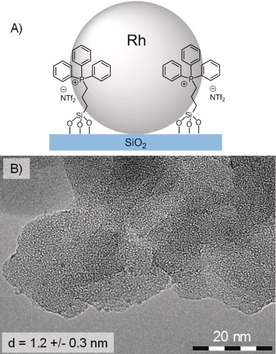
A) Illustration of the structure and B) transmission electron microscopy image of Rh@SILP(Ph_3_‐P‐NTf_2_).

Rhodium nanoparticles were generated on the SILP by impregnation with a solution of [Rh(allyl)_3_] in dichloromethane, followed by reduction of the dried and impregnated SILP under a hydrogen atmosphere (100 bar H_2_, 100 °C, 2 h) to give a black powder.16b The Rh loading on Rh@SILP(Ph_3_‐P‐NTf_2_) was determined to be 0.1 mmol g^−1^ by ICP‐AAS, in good agreement with the theoretical value. The BET surface area of the support decreased slightly from 292 to 271 m^2^ g^−1^ upon Rh loading (see Table S2 in the Supporting Information). Analysis of Rh@SILP(Ph_3_‐P‐NTf_2_) (illustrated schematically in Figure 2 A) by transmission electron microscopy showed that the NPs were small (1.2 nm) and well‐dispersed over the SILP support (Figure 2 B).

The catalytic activity of Rh@SILP(Ph_3_‐P‐NTf_2_) and several reference catalysts for hydrogenation and hydrodeoxygenation was first studied using acetophenone (**1**) as a model substrate (Table 1). The use of SiO_2_‐supported Rh NPs resulted in the hydrogenation of acetophenone (**1**) to form the saturated alcohol **1 a** in 95 % yield (entry 1). In contrast, Rh@SILP(Ph_3_‐P‐NTf_2_) produced the fully hydrodeoxygenated alkane **1 b** in quantitative yield (entry 2). Since neither Rh NPs nor phosphonium salts are known to catalyze the deoxygenation of ketones/alcohols individually, we investigated this intriguing reactivity by systematically modifying the catalyst structure.

**Table 1:** Hydrogenation and hydrodeoxygenation of acetophenone using Rh and Ru nanoparticles immobilized on various supports.^[a]^ 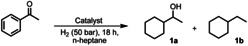

			Product yield [%]^[c]^	
Entry	Catalyst^[b]^	*T* [°C]	**1 a**	**1 b**
1	Rh@SiO_2_	100	95	5
2	Rh@SILP(Ph_3_‐P‐NTf_2_)	100	0	>99
3	Rh@SILP(Oct‐n_3_‐NTf_2_)	100	95	5
4^[d]^	Rh@SILP(Oct‐n_3_‐NTf_2_) + SILP(Ph_3_‐P‐NTf_2_)	100	97	3
5	Rh@SILP(Oct_3_‐P‐NTf_2_)	100	87	13
6	Rh@SILP(Ph_3_‐P‐BPh_4_)	100	>99	0
7	Rh@SILP(Ph_3_‐P‐BF_4_)	100	91	9
8	Ru@SILP(Ph_3_‐P‐NTf_2_)	100	89	11
9	Ru@SILP(Ph_3_‐P‐NTf_2_)	175	92	8

[a] Reaction conditions: catalyst (20 mg, metal content: 0.002 mmol), acetophenone (0.1 mmol, 50 equiv, 12.0 mg), n‐heptane (375 mg), H_2_ (50 bar), 18 h, 500 rpm. [b] See the Supporting Information for catalyst details. [c] Product distribution determined by GC‐FID using tetradecane as an internal standard, conversion >99 %. [d] SILP(Ph_3_‐P‐NTf_2_) (20 mg) was added (physical mixture).

With Rh NPs synthesized on an imidazolium‐based SILP (SILP(Oct‐n_3_‐NTf_2_), see the Supporting Information for details and Figure S9 for TEM characterization), the substrate was converted into the alcohol **1 a** (95 %) without any significant hydrodeoxygenation activity, thus suggesting that SILP(Ph_3_‐P‐NTf_2_) plays a key role in the deoxygenation step (entry 3). However, a physical mixture of Rh@SILP(Oct‐n_3_‐NTf_2_) and SILP(Ph_3_‐P‐NTf_2_) also gave **1 a** as the main product (97 %), showing the importance of the intimate contact of metal and support (entry 4). Using Rh NPs immobilized on a different phosphonium‐based SILP (Rh@SILP(Oct_3_‐P‐NTf_2_), see the Supporting Information for synthetic details and Figure S10 for TEM characterization) led to a mixture of products with the alcohol **1 a** as the main product (87 %; entry 5). Use of a fluorine‐free counteranion (BPh_4_, entry 6) resulted in the selective conversion of the substrate into the alcohol **1 a**. In the case of another fluorine‐containing anion (BF_4_, entry 7), no significant deoxygenation activity was observed, indicating that the deoxygenation‐active species was not formed. These results demonstrate the importance of the NTf_2_ anion for the observed deoxygenation activity. Finally, the metal precursor was changed to [Ru(cod)(cot)], and Ru NPs were then synthesized on SILP(Ph_3_‐P‐NTf_2_) (see the Supporting Information for synthetic details and Figure S11 for TEM characterization). The Ru@SILP(Ph_3_‐P‐NTf_2_) catalyst also produced the alcohol **1 a**, forming the hydrodeoxygenation product **1 b** only in low yield, even at high temperatures (11 % at 100 °C, 8 % at 175 °C; entries 8 and 9). Altogether, these results demonstrate that intimate contact between Rh nanoparticles and the SILP(Ph_3_‐P‐NTf_2_) support is necessary to create an efficient hydrodeoxygenation catalyst. A time profile was recorded (see Figure S12), showing fast conversion of the substrate in the first hour to give a mixture of **1 a** (54 %) and **1 b** (46 %). After that, **1 a** was gradually converted into **1 b**, producing the fully hydrodeoxygenated product in quantitative yield after 18 h.

**Figure 3:**
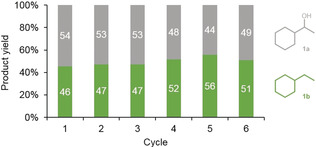
Product distribution for the hydrodeoxygenation of acetophenone using recycled Rh@SILP(Ph_3_‐P‐NTf_2_). The catalyst was washed with n‐heptane (1 mL) between cycles. Reaction conditions: catalyst (20 mg, metal content: 0.002 mmol Rh), acetophenone (12.0 mg, 0.1 mmol, 50 equiv), n‐heptane (375 mg), 100 °C, H_2_ (50 bar), 1 h, 500 rpm. Distribution was determined by GC‐FID using tetradecane as an internal standard. Green: ethylcyclohexane (**1 b**), gray: 1‐cyclohexylethanol (**1 a**). The conversion of the substrate was complete in all cases.

Recycling experiments showed that Rh@SILP(Ph_3_‐P‐NTf_2_) could be reused at least 5 times without any loss of activity or selectivity (Figure 3). For this assessment, the reaction conditions were intentionally tuned to yield a roughly 50:50 mixture of **1 a** and **1 b** in order to probe any change in performance. The catalyst could also be reused efficiently after 18 h reactions (see Table S1 in the Supporting Information).

No changes in the textural properties of the catalyst were evidenced by BET surface analysis (see Table S2). TEM characterization after catalysis suggests that the nanoparticles stay small and well‐dispersed on the support with only a slight increase of their size to 1.7 nm (see Figure S13). Elemental analysis by ICP‐AAS did not evidence any leaching of the metal during the reaction (see Table S2). Small amounts of IL were removed from the catalyst after the first cycle. After this initial loss, no further IL leaching was observed even after six consecutive experiments (see Table S2). ^1^H, ^13^C, and ^31^P solid‐state NMR spectroscopy of Rh@SILP(Ph_3_‐P‐NTf_2_) measured before and after catalysis showed partial hydrogenation of the phenyl groups of the phosphonium cation during Rh NP synthesis and catalysis (see Figures S5–S7). Interestingly, an additional peak at −122.5 ppm was observed in the ^19^F spectrum after catalysis (see Figure S8), indicating the partial conversion of the NTf_2_ anion into other F‐containing species. This signal has been previously ascribed to decomposition of the NTf_2_ anion with the formation of metal fluorides.^18^ No signal corresponding to silicon fluoride species^19^ were visible in the ^29^Si solid‐state NMR spectrum (see Figure S4). We thus suspect the formation of rhodium fluoride species through the partial breakdown of the NTf_2_ anion during the hydrogenation of the IL. The formation of acidic rhodium fluoride species at the surface of the nanoparticles could explain the hydrodeoxygenation activity of the catalyst. This hypothesis is consistent with the observation made by Forsyth and co‐workers, who observed the formation of magnesium fluoride species during the decomposition of the NTf_2_ anion under electrochemical conditions.^18^ Interestingly, thermal decomposition of the NTf_2_ anion is known to occur at high temperature (ca. 300 °C),^20^ but was observed in the present case at much lower temperature, thus indicating that the Rh nanoparticles promote the process. The absence of deoxygenation activity observed when using Ru as the metal suggests that the Ru NPs do not promote the anion decomposition under these conditions.

To gain more insight into the mechanism of formation of the active species—presumably rhodium fluoride—we carried out reference experiments. Heterogeneous catalysts are known to possess high hydrodefluorination activity, leading to the release of HF when hydrogenating fluoroaromatic substrates.^21^ Taking advantage of this reactivity, we performed a first reaction involving the hydrogenation of fluoroacetophenone with Rh@SiO_2_. This reaction gave a mixture of hydrogenated and hydrodefluorinated products, with the release of small quantities of HF (see Table S3). The catalyst was recovered, washed carefully, and characterized by ^19^F solid‐state NMR, which showed the appearance of a signal at −122.3 ppm, similar to that previously observed for the Rh@SILP(Ph_3_P‐NTf_2_) catalyst (see Figure S14). Furthermore, the application of this recycled catalyst to the conversion of acetophenone led to the formation of ethylcyclohexane, thus evidencing deoxygenation activity that the starting Rh@SiO_2_ did not possess (see Table S3). This result demonstrates that the deoxygenation activity is related to the formation of RhF species, which can be generated by the action of small quantities of HF on Rh nanoparticles. It supports the hypothesis that the RhF species observed on Rh@SILP(Ph_3_P‐NTf_2_) are formed through the decomposition of the NTf_2_ anion, a process which is known to generate HF among other products.^20^ The involvement of free HF in catalysis can, however, be ruled out, as a recycling experiment did not show any decrease in activity (Figure 3). Furthermore, the supernatant obtained after a catalytic reaction did not catalyze the deoxygenation of 1‐phenylethanol under standard conditions (see Table S1).

The hydrodeoxygenation activity of Rh@SILP(Ph_3_‐P‐NTf_2_) was further studied for a wide range of acetophenone derivatives with different steric and electronic properties (Table 2). All the substrates considered were effectively hydrodeoxygenated under mild reaction conditions, giving cyclohexane derivatives in high yields. The catalyst was found tolerant to various functional groups, even though partial ether cleavage and hydrodefluorination were observed for substrates **6** and **7**, respectively. In some cases, an increase in the temperature to 175 °C was necessary to reach full conversion (substrates **4** and **8**) or to limit the formation of dimers (substrates **9** and **10**). To get more insight into the influence of the presence of electron‐withdrawing and electron‐donating substituents on the catalytic activity, substrates **1**, **5**, **6**, and **8** were considered for a reaction time reduced to 1 h.

**Table 2:** Hydrodeoxygenation of substituted benzylic ketones using Rh@SILP(Ph_3_‐P‐NTf_2_).^[a]^ 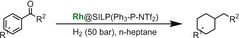

Entry	Substrate	*T* [°C]	*t* [h]	Product		Yield [%]^[b]^
1	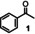	100 100	1 18	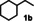		52^[c]^ >99
					
2	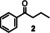	100	18	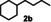		>99
					
3	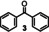	100	18	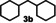		>99 (73)^[d]^
					
4	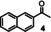	100 175	18 18	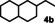		79^[c]^ >99
					
5	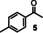	100 100	1 18	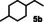		58^[c]^ >99
					
6	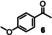	100 100 175	1 18 18	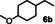	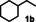	40 (**6 b**)^[e]^, 10 (**1 b**)^[e]^ 62 (**6 b**), 24^[f]^ (**1 b**) 25 (**6 b**), 75 (**1 b**)	
							
7	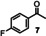	100	18	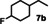	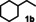	20 (**7 b**), 80 (**1 b**)	
							
8	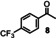	100 100 175	1 18 18	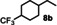		50^[c]^ 91^[c]^ >99	
			
9	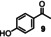	175	18	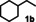		87 (**1 b**), 13 (dimers)	
						
10	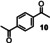	175	18	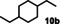		>99	

[a] Reaction conditions: Rh@SILP(Ph_3_‐P‐NTf_2_) (20 mg, metal content: 0.002 mmol Rh), substrate (0.1 mmol, 50 equiv), n‐heptane (375 mg), H_2_ (50 bar), 18 h, 500 rpm. [b] Determined by GC‐FID using tetradecane as an internal standard, conversion >99 %. [c] Remaining product: corresponding saturated alcohol. [d] The yield of the isolated product is given in brackets. [e] Total hydrodeoxygenation products: 71 %; 40 % **6 b**, 22 % 4‐ethyl‐cyclohexan‐1‐ol, 10 % **1 b**, 25 % 1‐(4‐methoxycyclohexyl)ethan‐1‐ol, 3 % cyclohexylethanol. [f] Remaining: 4‐ethyl‐cyclohexan‐1‐ol (10 %), cyclohexylethanol (3 %), (1‐methoxyethyl)cyclohexane (2 %).

**Scheme 1:**
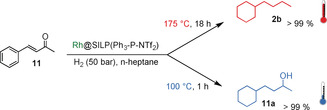
Temperature‐controlled conversion of benzylideneacetone (**11**) into either the fully hydrogenated product **11 a** or the completely hydrodeoxygenated product **2 b** using Rh@SILP(Ph_3_‐P‐NTf_2_).

Although electron‐donating substituents have been reported to have a positive effect on hydrodeoxygenation activity,^22^ we could not identify a clear trend in our particular case (see Table S4). Some of the substrates from Table 2 were then used to evaluate the catalytic activity of Ru@SILP(Ph_3_‐P‐NTf_2_) in comparison to Rh@SILP(Ph_3_‐P‐NTf_2_). In agreement with the preliminary results shown in Table 1 for acetophenone, the ruthenium‐based catalyst was barely active for the hydrodeoxygenation of the various aromatic ketones considered even at high temperature (see Tables S5 and S6). The catalytic properties of Rh@SILP(Ph_3_‐P‐NTf_2_) were further studied for the conversion of non‐benzylic ketones, which are known to be more challenging to hydrodeoxygenate than acetophenone derivatives.^23^ Benzylideneacetone (**11**) was used as a model substrate for this class of ketones (Scheme 1).

Keeping the conditions previously used for the hydrodeoxygenation of acetophenone derivatives led to almost exclusive formation of the saturated alcohol **11 a**. However, the conversion of **11** into the hydrodeoxygenated product **2 b** occurred with excellent selectivity at higher temperatures (175 °C). By adjusting the reaction temperature, both products (hydrogenated and hydrodeoxygenated) could be produced in quantitative yield. Again, the hydrodeoxygenation activity could not be switched on for Ru@SILP(Ph_3_‐P‐NTf_2_), and **11 a** was the only product at both 100 and 175 °C (see Table S6). To see whether such a “temperature switch” between hydrogenation and hydrodeoxygenation could be more generally applied to other substrates, we tested the conversion of a selection of non‐benzylic ketones at 100 and 175 °C using Rh@SILP(Ph_3_‐P‐NTf_2_) as the catalyst (Table 3). At 100 °C, substrates **11**–**16** (entries 1–6) possessing a phenyl ring in β or γ gamma position to the carbonyl group were converted into the saturated alcohols in high yields (81–99 %). Substrate **13**, however, was converted into a mixture of the saturated alcohol **13 a** (32 %) and partially hydrodeoxygenated product **11 a** (50 %).

**Table 3:** Temperature‐controlled hydrogenation or hydrodeoxygenation of non‐benzylic ketones using Rh@SILP(Ph_3_‐P‐NTf_2_).^[a]^ 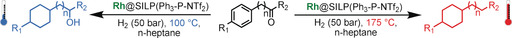

Entry	Substrate	Product 100 °C	Yield 100 °C [%]^[b]^	Product 175 °C		Yield 175 °C [%]^[b]^
1	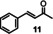	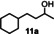	>99^[c]^ (87)^[d]^	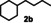		>99
					
2	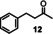	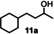	>99^[c]^	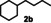		>99
					
3	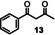	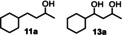	50 (**11 a**), 32^[e]^ (**13 a**)	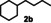		>99
					
4	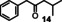	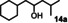	81^[f]^	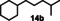		97
					
5	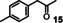	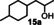	92	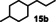		>99
					
6	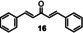	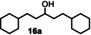	96 (92)^[d]^	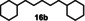		>99 (99)^[d]^
					
7	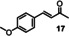	–	–	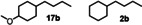		20 (**17 b**), 80 (**2 b**)
					
8	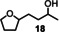	–	dimerization	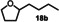	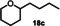	61 (**18 b**), 22 (**18 c**)^[g]^

[a] Reaction conditions: Rh@SILP(Ph_3_‐P‐NTf_2_) (20 mg, metal content: 0.002 mmol Rh), substrate (0.1 mmol, 50 equiv), n‐heptane (375 mg), 100 or 175 °C, H_2_ (50 bar), 18 h, 500 rpm. [b] Determined by GC‐FID using tetradecane as an internal standard, conversion >99 %. [c] Reaction time: 1 h. [d] The yield of the isolated product is given in brackets. [e] Determined by protection of the diol with benzaldehyde (see Scheme S1 in the Supporting Information). [f] 80 °C, 30 equiv. [g] Additional products: octanol (10 %), octane (7 %).

As expected, the hydrodeoxygenation of the benzylic alcohol was favored over the non‐benzylic alcohol, thus resulting in high amounts of **11 a** (50 %) as compared to 1‐cyclohexylbutanol (8 %). In the case of substrate **17**, only low mass balances were observed, indicating likely competing acid‐mediated condensation reactions of the enone substrate. All the substrates were efficiently hydrodeoxygenated at 175 °C to give the corresponding alkanes in excellent yields (97–99 %). In agreement with previous observations for substrate **6**, the methoxy group of substrate **17** was partially cleaved at 175 °C (entry 7). With biomass‐derived 4‐(tetrahydrofuran‐2‐yl)butan‐2‐ol (**18**, obtained from the complete hydrogenation of furfuralacetone) as the substrate, only various dimers were obtained when the reaction was carried out at 100 °C. However, the substrate was efficiently hydrodeoxygenated at 175 °C to produce a mixture of 2‐butyltetrahydrofuran (**18 b**), 2‐propyltetrahydro‐2*H*‐pyran (**18 c**), octanol, and octane (entry 8). Interestingly, these products are currently under discussion as potential alternative fuels and fuel additives.^24^

## Conclusion

In conclusion, we have shown that immobilizing Rh nanoparticles on a triphenylphosphonium‐based SILP produces a Rh@SILP(Ph_3_‐P‐NTf_2_) catalyst possessing excellent properties for the hydrogenation and hydrodeoxygenation of a wide range of aromatic ketones with various substituents. The required bifunctionality is enabled by a specific interaction between the Rh NPs and the SILP(Ph_3_‐P‐NTf_2_)) support, presumably leading to the formation of acidic Rh fluoride species. Using Rh@SILP(Ph_3_‐P‐NTf_2_), acetophenone derivatives were readily hydrodeoxygenated under mild conditions. For non‐benzylic ketones, the product distribution could be switched with high selectivity between the hydrogenated and hydrodeoxygenated products simply by tuning the temperature. The flexibility and modularity of the molecular approach used to prepare NPs@SILP catalysts allowed for the assembly of the exact key components required to achieve this unique reactivity. This approach opens the way to the efficient production of highly valuable cyclohexane derivatives from readily available aromatic ketones.

## Conflict of interest

The authors declare no conflict of interest.

## Supporting information

As a service to our authors and readers, this journal provides supporting information supplied by the authors. Such materials are peer reviewed and may be re‐organized for online delivery, but are not copy‐edited or typeset. Technical support issues arising from supporting information (other than missing files) should be addressed to the authors.

Supplementary
