# Supplementary material for: Selective Hydrogenation and Hydrodeoxygenation of Aromatic Ketones to Cyclohexane Derivatives Using a Rh@SILP Catalyst
Source: Angew Chem Int Ed Engl. 2020 May 27;59(29):11977–83. doi: 10.1002/anie.201916385 (PMC7383641; doi:10.1002/anie.201916385)
Supplement: Supplementary file 1 — Supplementary [file ANIE-59-11977-s001.pdf]

Angewandte Chemie
Eine Zeitschrift der Gesellschaft Deutscher Chemiker

Supporting Information

Selective Hydrogenation and Hydrodeoxygenation of Aromatic Ketones to Cyclohexane Derivatives Using a Rh@SILP Catalyst

Gilles Moos, Meike Emondts, Alexis Bordet, and Walter Leitner**

anie_201916385_sm_miscellaneous_information.pdf

Supporting Information:

Safety Warning:

High pressure experiments with compressed H_2 must be carried out only with appropriate equipment and under rigorous safety precautions.

General

If not otherwise stated, the synthesis of the ionic liquids (ILs), the supported ionic liquid phases (SILPs) and the nanoparticles immobilized on SILPs (NPs@SILPs) were carried out under an inert atmosphere (Ar) using standard Schlenk techniques or inside a glovebox. All the synthesized materials were stored under inert atmosphere except from the Rh@SILP. Benzylideneacetone (**11**) was purified by sublimation prior to use. [Rh(allyl)$_3$] was synthesized according to a modified literature procedure.[1] All other chemicals and solvents were commercially available and used without further purification.

Synthesis of NP@SILP catalysts

Ionic liquid synthesis:

Synthesis of triphenyl(3-(triethoxysilyl)propyl)phosphonium iodide:

A mixture of triphenylphosphine (6.03 g, 23 mmol, 1 eq.) and triethoxy(3-iodopropyl)silane (7.64 g, 23 mmol, 1 eq.) in 30 mL anhydrous toluene was refluxed over night at 130 °C under inert atmosphere. After the reaction mixture cooled down, a phase separation was observable. The toluene phase was removed and the IL was washed two times with dry toluene and n-pentane. The product, a white solid, was dried over night at 60 °C in vacuo. Yield = 78%.

^{1}H NMR (400 MHz, (CD3)$_2$CO, δ): 0.97 (t, 2H, *J* = 8.0 Hz), 1.12 (t, 9H, *J* = 7.0 Hz), 1.80-1.92 (m, 2H), 3.70-3.80 (m, 8H, 2 peaks overlaying), 7.75-8.03 (m, 15H).

^{13}C NMR (100 MHz, (CD3)$_2$CO, δ): 136.08 (d, *J* = 3.0 Hz), 135.8 (d, *J* = 10.0 Hz), 131.4 (d, *J* = 12.3 Hz), 119.8 (d, *J* = 87.5 Hz), 59.1, 25.1 (d, *J* = 47.8 Hz), 18.8, 17.8 (d, *J* = 4.0 Hz), 12.4 (d, *J* = 16.0 Hz)

^{31}P NMR (162 MHz, (CD3)$_2$CO, δ): 23.10 (s, 1P), 23.25 (impurities).

Synthesis of triphenyl(3-(triethoxysilyl)propyl)phosphonium bis((trifluoromethyl)sulfonyl)amide:

For the subsequent anion exchange no Schlenk techniques were used. Triphenyl(3-(triethoxysilyl)propyl)phosphonium iodide (3.0 g, 5.1 mmol, 1 eq.) was dissolved in 7 mL DCM. In a separate flask, $LiNTf_2$ (1.5 g, 5.1 mmol, 1 eq.) was dissolved in 1.5 mL water. The solutions were mixed and vigorously stirred for 30 min at room temperature. The organic phase was washed three times with water, dried over $MgSO_4$ and the solvent was removed under reduced pressure. The resulting pale viscous liquid was dried over night at 60 °C in vacuo. Yield = 90%. (Abbreviation: Ph_3-P-NTf_2).

^{1}H NMR (400 MHz, (CD3)$_2$CO, δ): 0.94 (t, 2H, *J* = 8.0 Hz), 1.13 (t, 9H, *J* = 7.0 Hz), 1.82-1.94 (m, 2H), 3.57-3.66 (m, 2H), 3.77 (q, 6H, *J* = 7.0 Hz) 7.80-7.99 (m, 15H).

^{13}C NMR (100 MHz, (CD3)$_2$CO, δ): 12.28 (d, *J* = 16.0 Hz), 17.61 (d, *J* = 4.0 Hz), 18.58 (s), 24.68 (d, *J* = 48.0 Hz), 58.94 (s), 119.42 (d, *J* = 86.1 Hz), 131.24 (d, *J* = 12.7 Hz), 134.51 (d, *J* = 9.95 Hz), 136.0 (d, *J* = 3.0 Hz).

^{31}P NMR (162 MHz, (CD3)$_2$CO, δ): 22.93 (s, 1P), 22.81 (s, impurity small).

^{19}F NMR (376 MHz, (CD3)$_2$CO, δ): -79.70 (s, 1F).

HRMS/ESI(+) (CH_2Cl_2): m/z = 467.215990 calcd. for $[C_{27}H_{36}O_3PSi]^+$ = 467.216588

Synthesis of triphenyl(3-(triethoxysilyl)propyl)phosphonium tetraphenylborate:

For the subsequent anion exchange no Schlenk techniques were used. Triphenyl(3-(triethoxysilyl)propyl)phosphonium iodide (2.0 g, 3.4 mmol, 1 eq.) was dissolved in 7 mL DCM. In

a separate flask, $NaBPh_4$ (1.3 g, 3.7 mmol, 1.1 eq.) was dissolved in 1.5 mL water. The solutions were mixed and vigorously stirred for 30 min at room temperature. The organic phase was washed three times with water, dried over $MgSO_4$ and the solvent was removed under reduced pressure. The resulting white solid was dried over night at 60 °C in vacuo. (Abbreviation: Ph_3-P-BPh_4).

^{1}H NMR (400 MHz, $(CD3)_2CO$, δ): 0.90 (t, 2H, *J* = 8.0 Hz), 1.13 (t, 9H, *J* = 7.0 Hz), 1.81-1.91 (m, 2H), 3.51-3.60 (m, 2H), 3.77 (q, 6H, *J* = 7.0 Hz), 6.77 (t, 4H, *J* = 7.2 Hz), 6.91 (t, 8H, *J* = 7.5 Hz), 7.31-7.37 (m, 8H), 7.80-7.99 (m, 15H).

^{13}C NMR (100 MHz, $(CD3)_2CO$, δ): 11.54 (d, *J* = 16.2 Hz), 16.77 (d, *J* = 4.2 Hz), 17.78 (s), 23.90 (d, *J* = 49.2 Hz), 58.21 (s), 118.56 (d, *J* = 87.3 Hz), 121.36 (s), 125.11 (q, *J* = 2.8 Hz), 130.43 (d, *J* = 12.2 Hz), 133.68 (d, *J* = 9.8 Hz), 135.19 (d, *J* = 3.2 Hz), 136.16 (q, *J* = 1.4 Hz), 164.06 (q, *J* = 49.4 Hz).

^{31}P NMR (162 MHz, $(CD3)_2CO$, δ): 22.89 (s, 1P).

^{11}B NMR (128 MHz, $(CD3)_2CO$, δ): -6.52 (s, 1B).

<u>Synthesis of triphenyl(3-(triethoxysilyl)propyl)phosphonium tetrafluoroborate:</u>

For the subsequent anion exchange no Schlenk techniques were used. Triphenyl(3-(triethoxysilyl)propyl)phosphonium iodide (2.0 g, 3.4 mmol, 1 eq.) was dissolved in 7 mL DCM. In a separate flask, $NaBF_4$ (0.4 g, 3.7 mmol, 1.1 eq.) was dissolved in 1.0 mL water. The solutions were mixed and vigorously stirred for 30 min at room temperature. The organic phase was washed three times with water, dried over $MgSO_4$ and the solvent was removed under reduced pressure. The resulting white solid was dried over night at 60 °C in vacuo. (Abbreviation: Ph_3-P-BF_4).

^{1}H NMR (400 MHz, $(CD3)_2CO$, δ): 0.96 (t, 2H, *J* = 8.0 Hz), 1.12 (t, 9H, *J* = 7.0 Hz), 1.81-1.91 (m, 2H), 3.67-3.73 (m, 2H), 3.77 (q, 6H, *J* = 7.0 Hz), 6.77 (t, 4H, *J* = 7.2 Hz), 6.91 (t, 8H, *J* = 7.5 Hz), 7.80-7.85 (m, 6H), 7.93-7.99 (m, 9H).

^{13}C NMR (100 MHz, $(CD3)_2CO$, δ): 12.38 (d, *J* = 16.0 Hz), 17.73 (d, *J* = 4.0 Hz), 18.71 (s), 24.85 (d, *J* = 49.0 Hz), 59.05 (s), 119.70 (d, *J* = 85.8 Hz), 131.35 (d, *J* = 12.0 Hz), 134.74 (d, *J* = 10.0 Hz), 136.10 (d, *J* = 3.0 Hz).

^{31}P NMR (162 MHz, $(CD3)_2CO$, δ): 23.06 (s, 1P).

^{19}F NMR (376 MHz, $(CD3)_2CO$, δ): -151.77 (s, F).

<u>Synthesis of 1-octyl-3-(3-(triethoxysilyl)propyl)-1*H*-imidazol-3-ium bromide</u>

3-(3-triethoxysilylpropyl)imidazole (10.1 g, 37.1 mmol) and octyl bromide (8.9 g, 46.3 mmol) were dissolved in toluene (50 mL) and stirred under reflux at 130 °C for 18 h. The resulting mixture was washed 3 times with n-pentane (3x40 mL). The product was dried *in vacuo* to yield a viscous, yellowish liquid (4.86 g, 90%). (Abbreviation: Oct-n_3-Br)

^{1}H NMR (300 MHz, $(CD3)_2CO$, δ): 10.14 (s, 1H, NCHN), 7.90 (m, 2H, NCHCHN), 4.45 (m, 4H, NCH_2), 3.82 (q, *J* = 7.0 Hz, 6H, OCH_2), 2.01-1.68 (m, 4H, CH_2), 1.36 (m, 10H, CH_2), 1.19 (t, *J* = 7.0 Hz, 9H, OCH_2CH_3), 0.87 (t, *J* = 7.3 Hz, 3H, CH_3), 0.63 (m, 2H, $SiCH_2$)

Synthesis of 1-Octyl-3-(3-triethoxysilylpropyl)imidazolium bis(trifluoromethylsulfonyl)imide:

1-octyl-3-(3-triethoxysilylpropyl)imidazolium bromide (5.6 g, 12.0 mmol) and bis(trifluoromethane)sulfonimide lithium salt (3.6 g, 12.6 mmol) were dissolved in water (20 mL) and stirred at rt for 1 h. DCM (50 mL) was added and the organic phase was washed with water (3x50 mL). The organic phase was dried over $MgSO_4$ and the solvent was removed under reduced pressure. The product was dried *in vacuo* to yield a viscous, yellow/brown liquid (6.72 g, 84%). (Abbreviation: Oct-n_3-NTf_2)

^{1}H NMR (300 MHz, (CD3)$_2$CO, δ): 9.14 (s, 1H, NCHN), 7.83 (m, 2H, NCHCHN), 4.38 (m, 4H, NCH_2), 3.82 (q, J = 7.0 Hz, 6H, OCH_2), 2.11-1.94 (m, 4H, CH_2), 1.36 (m, 10H, CH_2), 1.18 (t, J = 7.0 Hz, 9H, OCH_2CH_3), 0.87 (t, J = 7.3 Hz, 3H, CH_3), 0.62 (m, 2H, $SiCH_2$).

^{13}C NMR (150 MHz, (CD3)$_2$CO, δ): 136.8 (s, 1C, NCHN), 123.7 (s, 1C, NCHCHN), 123.6 (s, 1C, NCHCHN), 119.2 (q, J_{CF} = 327 Hz, 2C, CF_3), 59.0 (s, 3C, OCH_2), 52.8 (s, 1C, NCH_2), 50.7 (s, 1C, NCH_2), 32.5 (s, 1C, CH_2), 30.7 (s, 1C, CH_2), 29.8 (s, 1C, CH_2), 29.1 (s, 1C, CH_2), 27.3 (s, 1C, CH_2), 26.9 (s, 1C, CH_2), 25.0 (s, 1C, CH_2), 23.3 (s, 1C, CH_2), 18.7 (s, 3C, OCH_2CH_3), 14.3 (s, 1C, CH_3), 7.8 (s, 1C, $SiCH_2$).

HRMS/ESI(+) (CH_2Cl_2): m/z = 385.288096 and 279.917850, calcd for $[C_{20}H_{41}N_2O_3Si]^+$ = 385.288160 and $[C_2F_6N_1O_4S_2]^-$ = 279.917920.

Synthesis of trioctyl(3-(triethoxysilyl)propyl)phosphonium iodide

A mixture of trioctylphosphine (8.52 g, 23 mmol, 1 eq.) and triethoxy(3-iodopropyl)silane (7.64 g, 23 mmol, 1 eq.) in 30 mL anhydrous toluene was refluxed over night at 130 °C under inert atmosphere. After the reaction mixture cooled down, a phase separation was observable. The toluene phase was removed and the IL was washed two times with dry toluene and n-pentane. The product, a pale viscous liquid, was dried over night at 60 °C in vacuo. Yield = 92%. (Abbreviation: Oct_3-P-I)

^{1}H NMR (300 MHz, $CDCl_3$, δ): 3.84 (m, 6H, OCH_2), 2.53 (m, 8H, PCH_2), 1.90-1.31 (m, 38H, CH_2), 1.21 (m, 9H, CH_3), 0.89 (m, 11H, CH_3, $SiCH_2$).

^{31}P NMR (162 MHz, $CDCl_3$, δ): 31.76 (m, 1P).

Synthesis of trioctyl(3-(triethoxysilyl)propyl)phosphonium NTf_2

For the subsequent anion exchange no Schlenk techniques were used. Trioctyl(3-(triethoxysilyl)propyl)phosphonium iodide (7.0 g, 10 mmol, 1eq) was dissolved in 15 mL DCM. In a separate flask, $LiNTf_2$ (2.87 g, 10 mmol, 1eq) was dissolved in 2.5 mL water. The solutions were mixed and vigorously stirred for 30 min at room temperature. The organic phase was washed three times with water, dried over $MgSO_4$ and the solvent was removed under reduced pressure. The resulting pale viscous liquid was dried over night at 60 °C in vacuo. Yield = 85%. (Abbreviation: Oct_3-P-NTf_2)

^{1}H NMR (300 MHz, (CD3)$_2$CO, δ): 3.84 (m, 6H, OCH_2), 2.48 (m, 8H, PCH_2), 1.73-1.30 (m, 38H, CH_2), 1.21 (m, 9H, CH_3), 0.89 (m, 11H, CH_3, $SiCH_2$).

^{13}C NMR (100 MHz, (CD3)$_2$CO, δ): 59.02 (s, 3C), 32.51 (s, 3C), 29.73 (s, 3C), 29.53 (s, 3C), 23.07 (s, 3C), 22.07 (s, 3C), 22.01 (s, 3C), 19.48 (s, 1C), 18.85 (s, 1C), 18.73 (s, 3C), 14.34 (s, 3C), 12.51 (s, 1C).

^{31}P NMR (162 MHz, (CD3)$_2$CO, δ): 32.86 (m, 1P).

SILP synthesis:

General procedure for the synthesis of SILP

A solution of the ionic liquid (9.0 mmol) in 20 mL anhydrous DCM was added to a suspension of 10.3 g dehydroxylated silica (500 °C, high vacuum, 16 h) in 90 mL anhydrous toluene. The reaction mixture was refluxed at 130 °C for 2-5 days under inert atmosphere, before the solvent was carefully removed by decantation. The SILP was washed 4 times with dry DCM and dried in vacuo at 60 °C for 16 h. The solvent of the combined organic phases was removed under reduced pressure to determine the amount of not grafted IL. (Total IL grafted = starting amount of IL – recovered IL).[2]

NPs@SILP synthesis:

Synthesis of Rh@SILP

A solution of [Rh(allyl)$_3$] (11.3 mg, 0.05 mmol) in 2 mL dry DCM was added to a suspension of 500 mg support (SILP or SiO_2) in 5 mL dry DCM. The reaction mixture was stirred for 1 h at room temperature and the support changed its color to yellow/brown. The solvent is carefully removed under reduced pressure and the impregnated support material was transferred to an autoclave within the glovebox. The metal precursor was reduced under the following conditions: 100 °C, 100 bar H_2 (at r.t), 2h. A grey/black powder was obtained and stored under air.

Synthesis of Ru@SILP

A solution of [Ru(cod)(cot)] (15.7 mg, 0.05 mmol) in 2 mL dry DCM was added to a suspension of 500 mg SILP in 5 mL dry DCM. The reaction mixture was stirred for 1 h at room temperature and the SILP changed its color grey. The solvent is carefully removed under reduced pressure and the impregnated SILP was transferred to an autoclave within the glovebox. The metal precursor was reduced under the following conditions: 150 °C, 20 bar H_2 (at r.t), 1h. A grey/black powder was obtained and stored under inert atmosphere.

Catalytic reactions

All high pressure reactions were carried out using in-house manufactured 10 or 20 mL stainless steel finger autoclaves. Catalytic reactions were performed in glass inlets.

The substrate (0.1 mmol, 50 eq.), the catalyst (20 mg, 0.002 mmol metal) and n-heptane (375 mg) were mixed in a glass inlet and placed in a high pressure autoclave. The reactor was purged (5 times) and pressurized with H_2, before it was brought to reaction temperature using a pre-heated aluminum cone. Mixing was guaranteed by using a magnetic stirrer bar at 500 rpm. The reactions were stopped by quickly cooling the autoclave in a water-bath to room temperature and careful depressurization. The reaction mixture was filtered using a syringe filter and the solution was analyzed by GC-FID using tetradecane as an internal standard.

For the recycling experiments, the catalyst was separated via centrifugation from the solution. The solution was submit to GC analysis and the catalyst was washed with n-heptane (1mL) before a fresh substrate solution was added and the reaction restarted under the same conditions.

Analytics

All solution state NMR were recorded on a Bruker Ascend 400 spectrometer at room temperature. The coupling constants (*J*) are given in Hertz (Hz), and the chemical shifts (*δ*) are expressed in ppm, relative to TMS at 25 °C. Gas chromatography (GC) was performed on a Shimadzu GC-2030 equipped with a FID-detector and a CP-WAX-52CB column from Agilent. Gas chromatography coupled with a mass spectrometer (GC-MS) were performed on a Shimadzu QP2020. All TEM images were recorded on a Hitachi HF2000 operating at 200 kV. Elemental Analysis was measured externally in “Mikroanalytisches Laboratorium Kolbe, Oberhausen Germany”. BET measurements were performed on a Quadrasorb SI from Quantachrom Instruments. FTIR spectra of SILPs and dehydroxylated SiO_2 were obtained using a Bruker Alpha spectrometer in the DRIFT mode and ^{29}Si solid-state NMR spectra were obtained using a Bruker AVIII-500 spectrometer.

Results and Discussion

Catalysts characterization

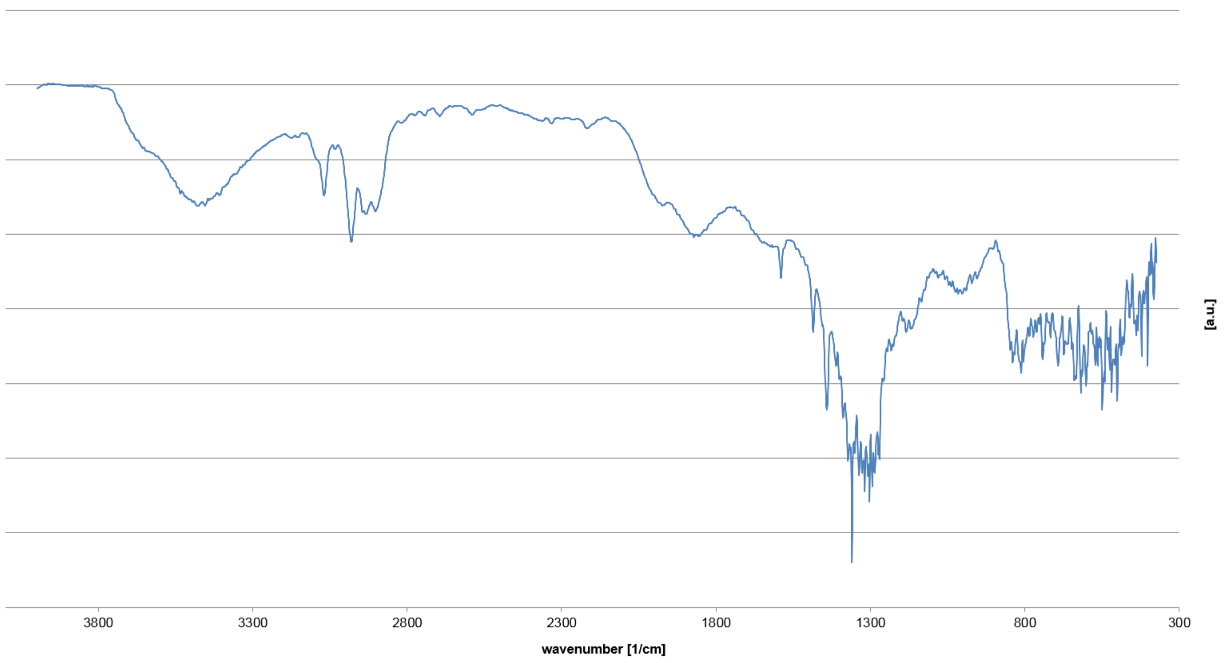

Figure S1: Infrared spectrum (DRIFT) of SILP(Ph_3-P-NTf_2).

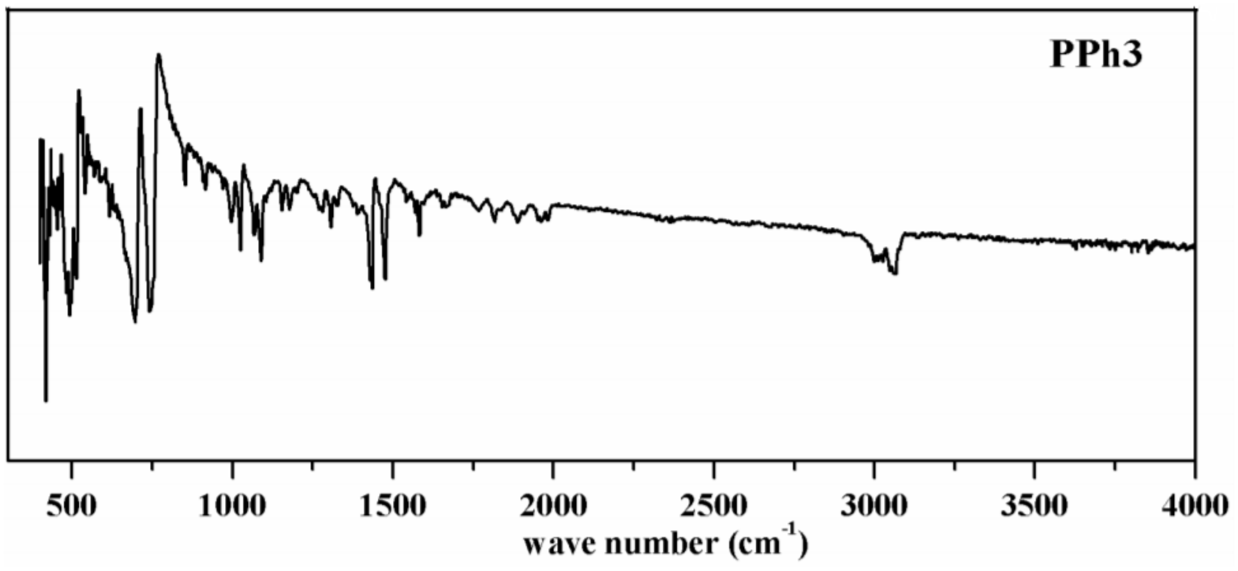

Figure S2: FT-IR of triphenylphosphine.[3]

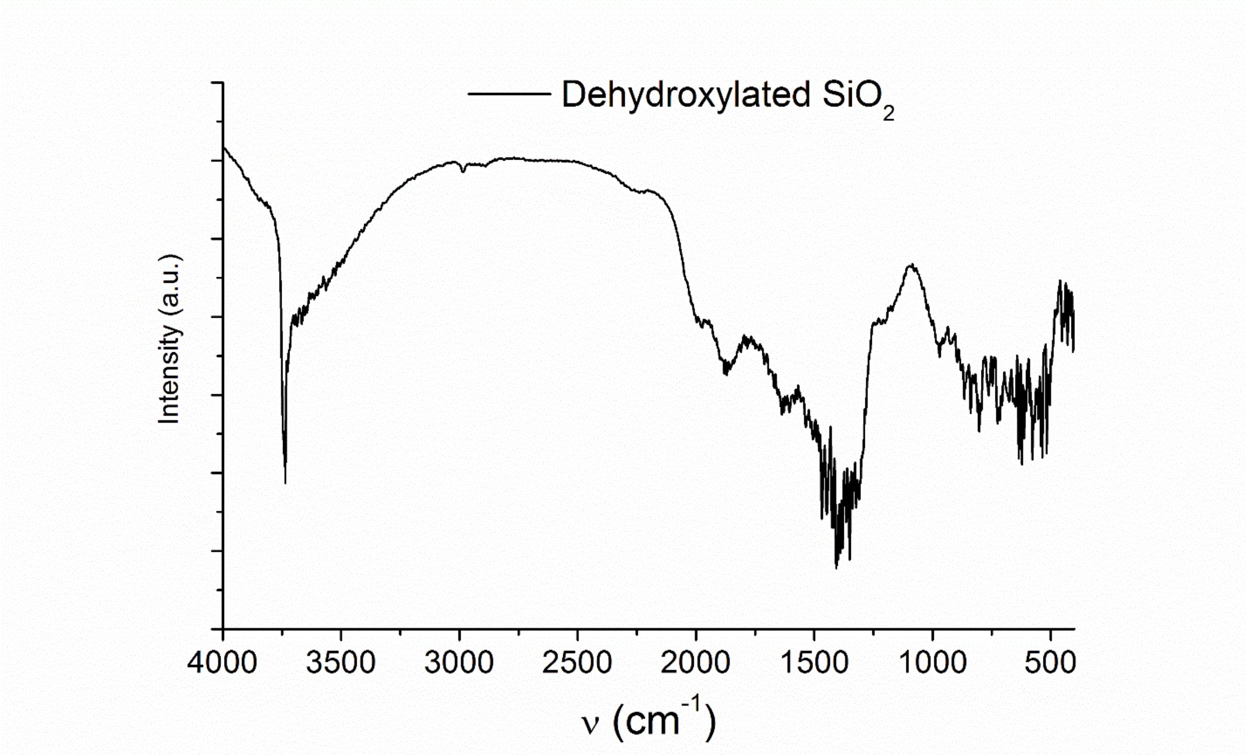

Figure S3: Infrared spectrum (DRIFT) of dehydroxylated silica.

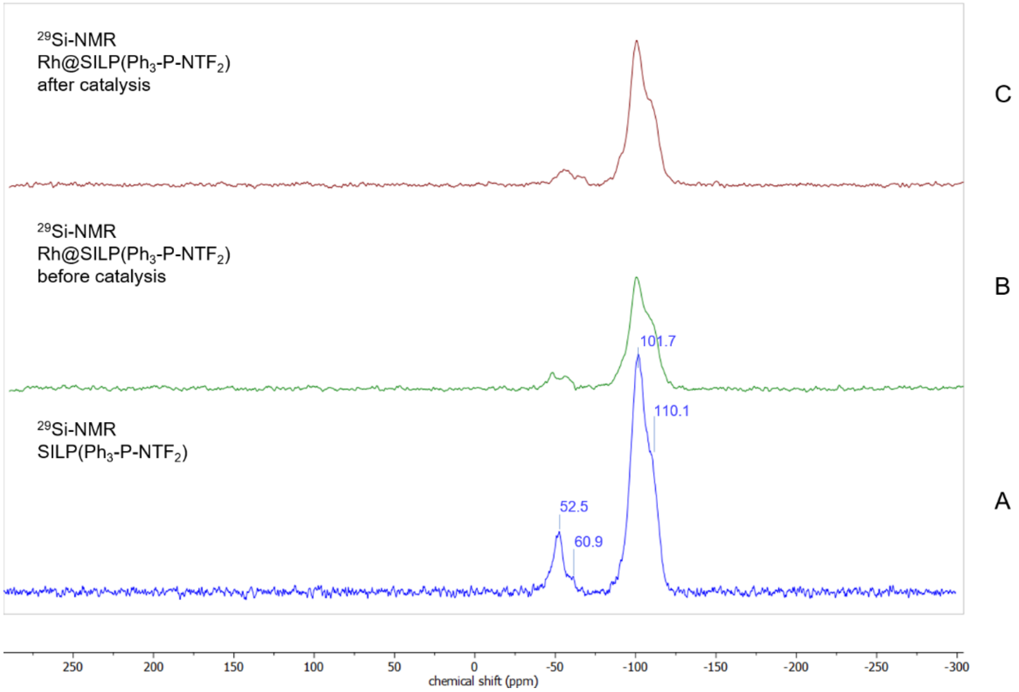

Figure S4: CP-MAS Solid state NMR (^{29}Si) of SILP(Ph_3-P-NTf_2) (A, blue), Rh@SILP(Ph_3-P-NTf_2) before catalysis (B, green) and Rh@SILP(Ph_3-P-NTf_2) after catalysis (C, red).

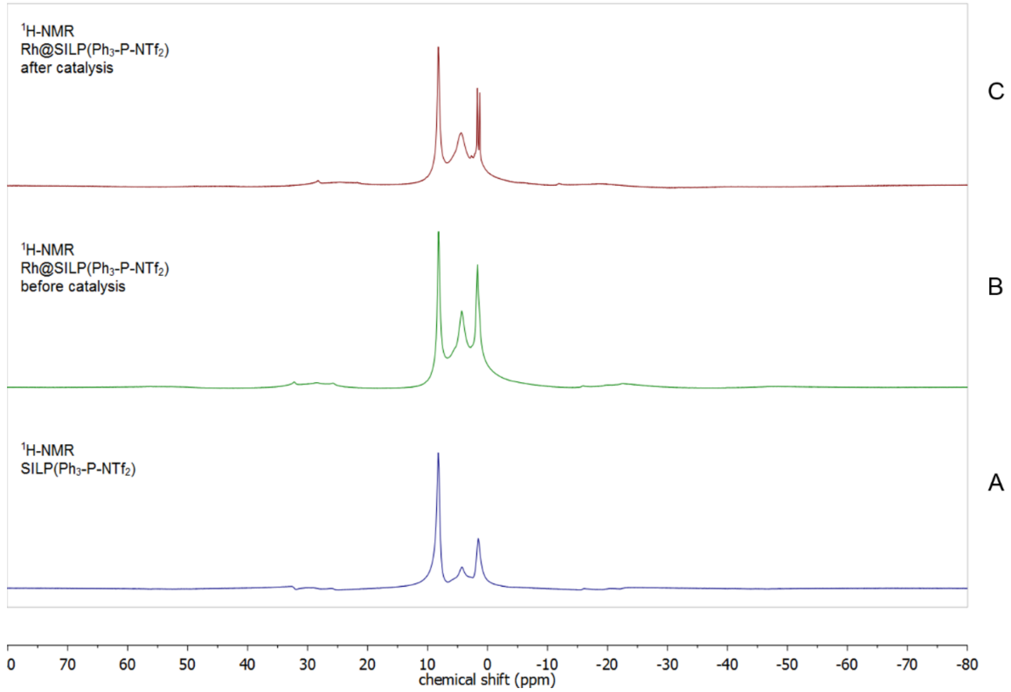

Figure S5: CP-MAS Solid state NMR (^{1}H) of SILP(Ph_3-P-NTf_2) (A, blue), Rh@SILP(Ph_3-P-NTf_2) before catalysis (B, green) and Rh@SILP(Ph_3-P-NTf_2) after catalysis (C, red).

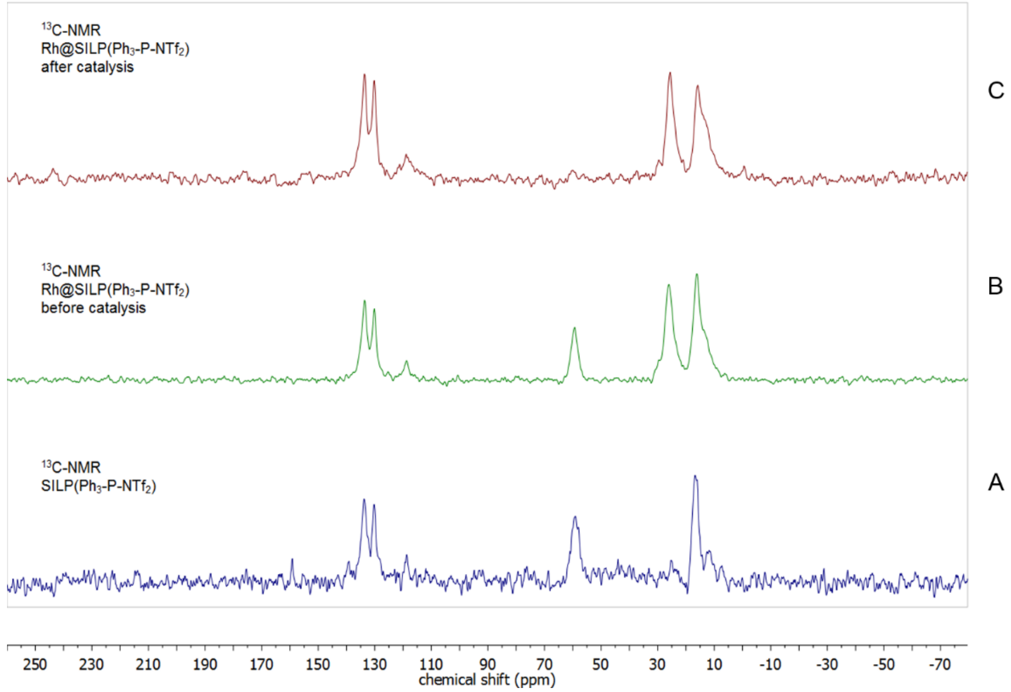

Figure S6: CP-MAS Solid state NMR (^{13}C) of SILP(Ph_3-P-NTf_2) (A, blue), Rh@SILP(Ph_3-P-NTf_2) before catalysis (B, green) and Rh@SILP(Ph_3-P-NTf_2) after catalysis (C, red).

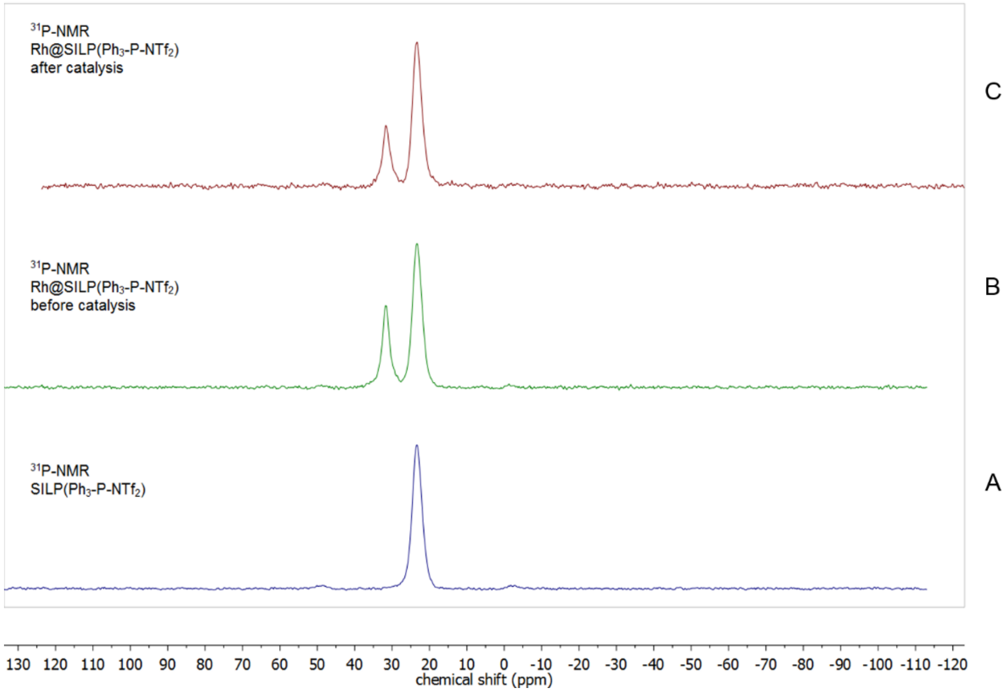

Figure S7: CP-MAS Solid state NMR (^{31}P) of SILP(Ph_3-P-NTf_2) (A, blue), Rh@SILP(Ph_3-P-NTf_2) before catalysis (B, green) and Rh@SILP(Ph_3-P-NTf_2) after catalysis (C, red).

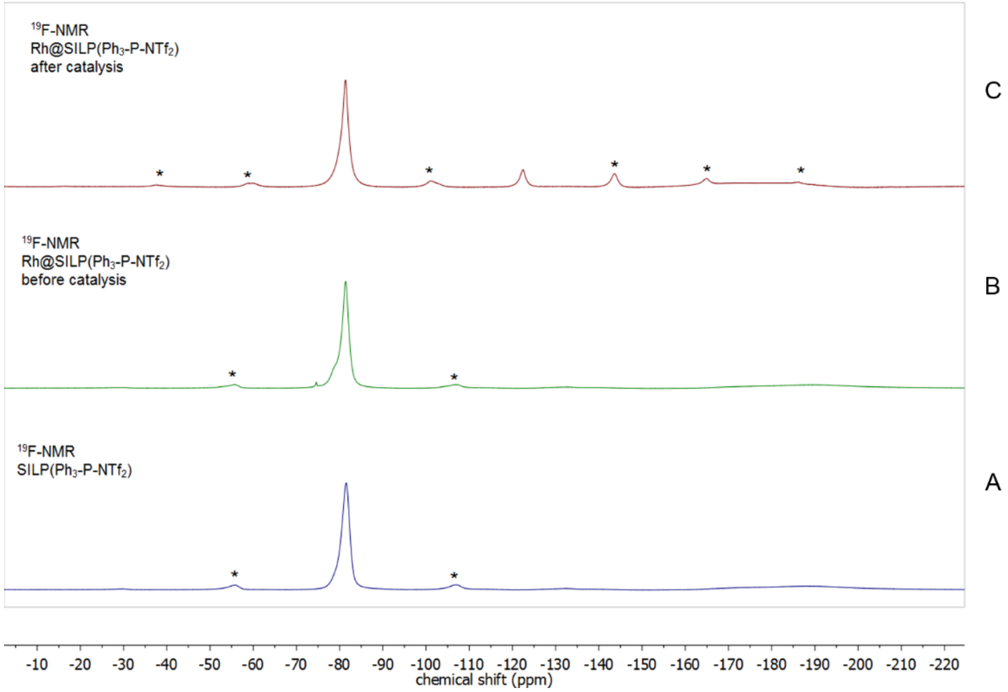

Figure S8: CP-MAS Solid state NMR (^{19}F) of SILP(Ph_3-P-NTf_2) (A, blue), Rh@SILP(Ph_3-P-NTf_2) before catalysis (B, green) and Rh@SILP(Ph_3-P-NTf_2) after catalysis (C, red). Rotational sidebands marked by *.

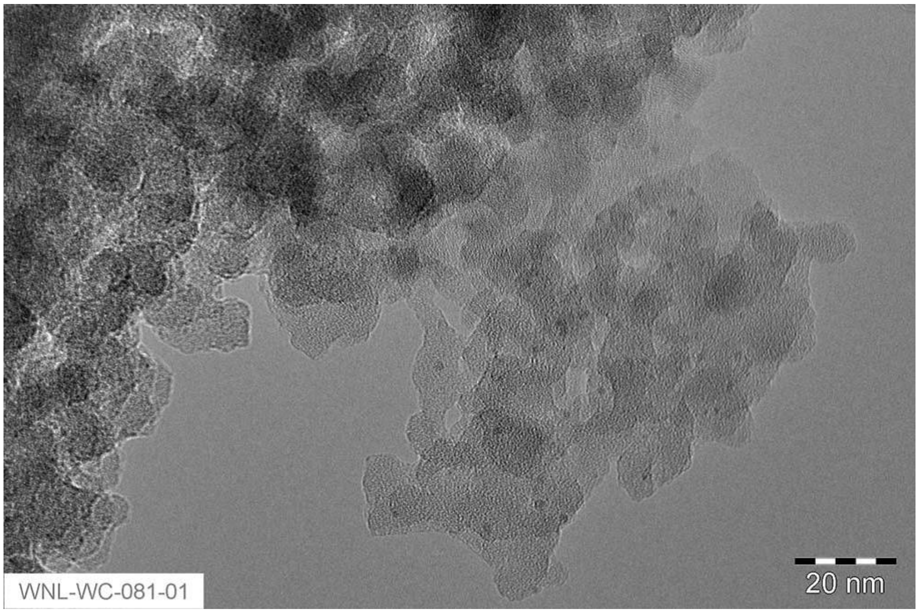

Figure S9: Transmission electron microscopy (TEM) image of Rh@SILP(Oct-n_3-NTf_2) with a particle size of 1.2 +/- 0.3 nm

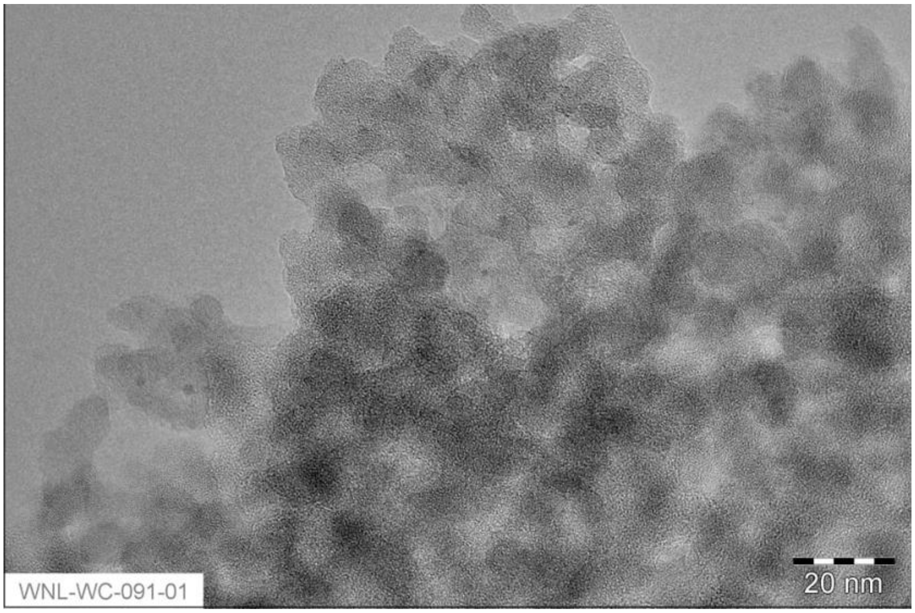

Figure S10: Transmission electron microscopy (TEM) image of Rh@SILP(Oct_3-P-NTf_2) with a particle size of 1.4 +/- 0.5 nm.

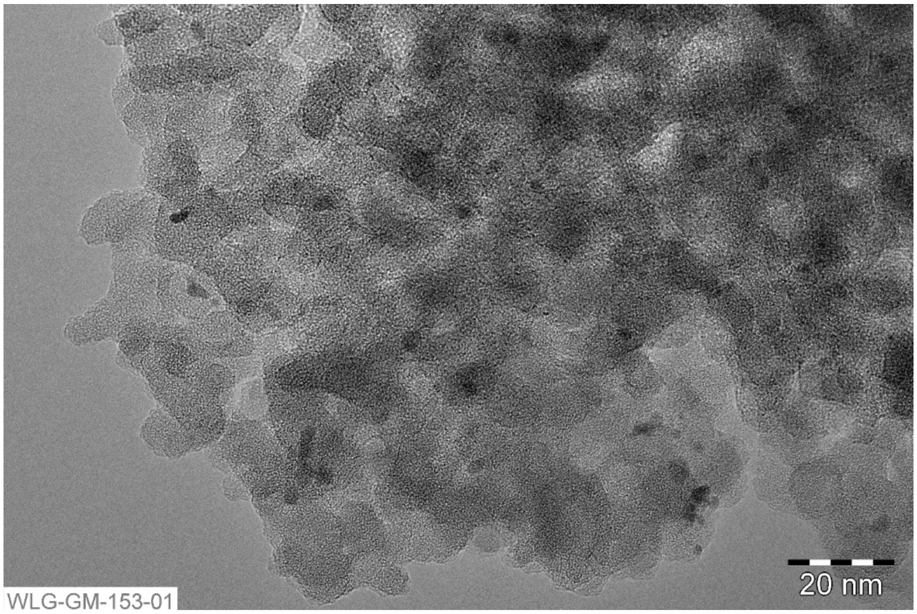

Figure S11: Transmission electron microscopy (TEM) images of Ru@SILP(Ph_3-P-NTf_2) with a particle size of 1.8 +/- 0.3 nm.

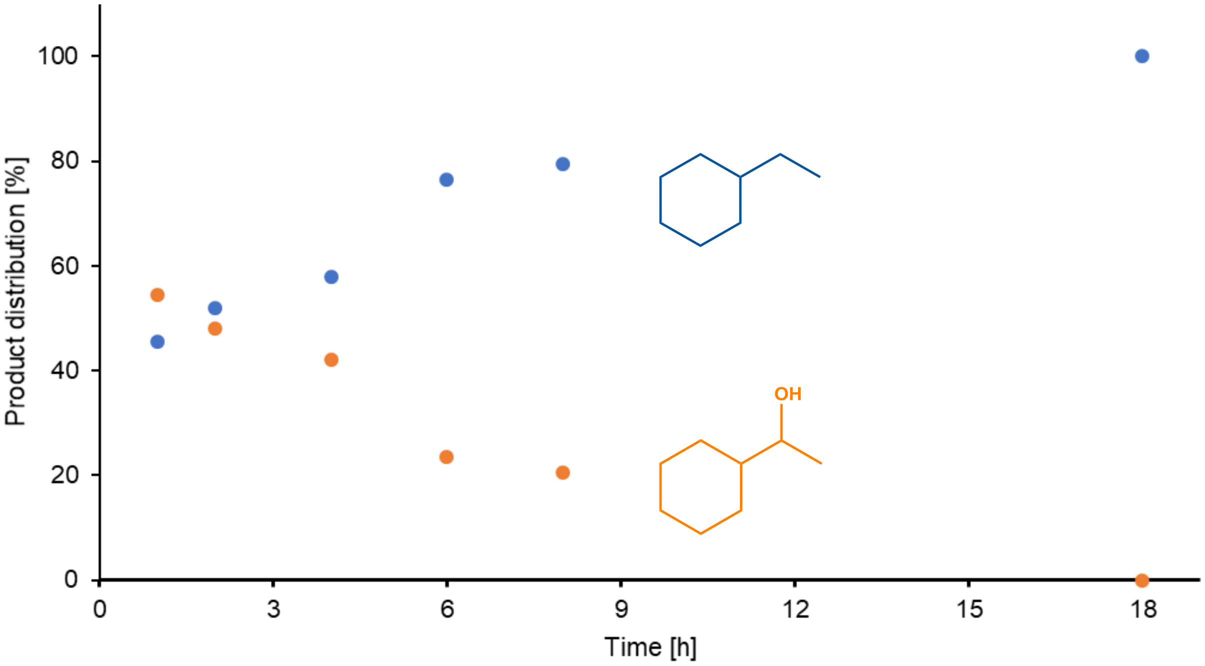

Figure S122: Time profile for the conversion of acetophenone using Rh@SILP(Ph_3-P-NTf_2). Reaction conditions: Cat (20 mg, metal content: 0.002 mmol Rh), acetophenone (12.0 mg, 0.1 mmol, 50 eq.), n-heptane (375 mg), 100 °C, H_2 (50 bar), 500 rpm. Reaction time: 1h, 2h, 4h, 6h, 8h, 18h. Substrate conversion = 100% for all points.

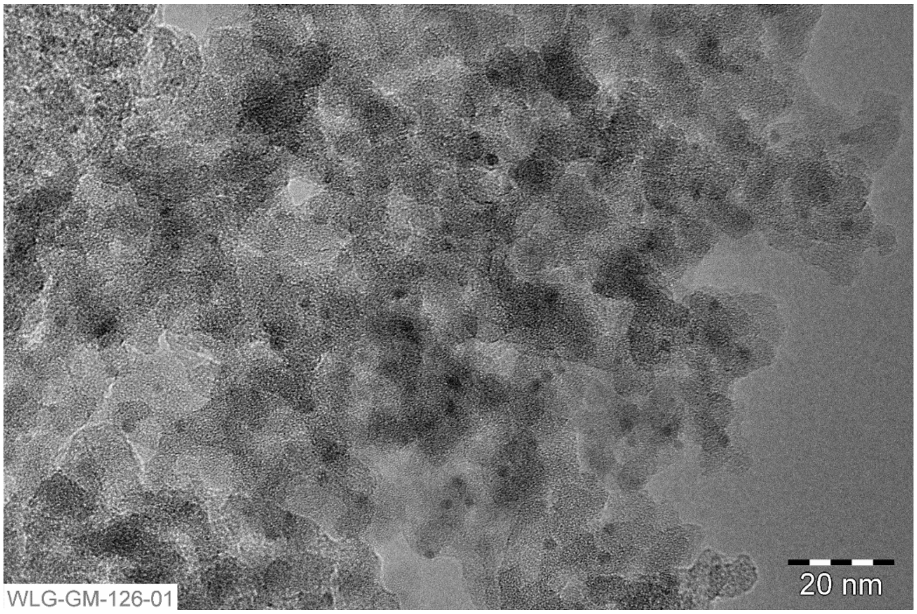

Figure S133: Transmission electron microscopy (TEM) image of Rh@SILP(Ph_3-P-NTf_2) after recycling experiment (6 runs) with a particle size of 1.7 +/- 0.3 nm.

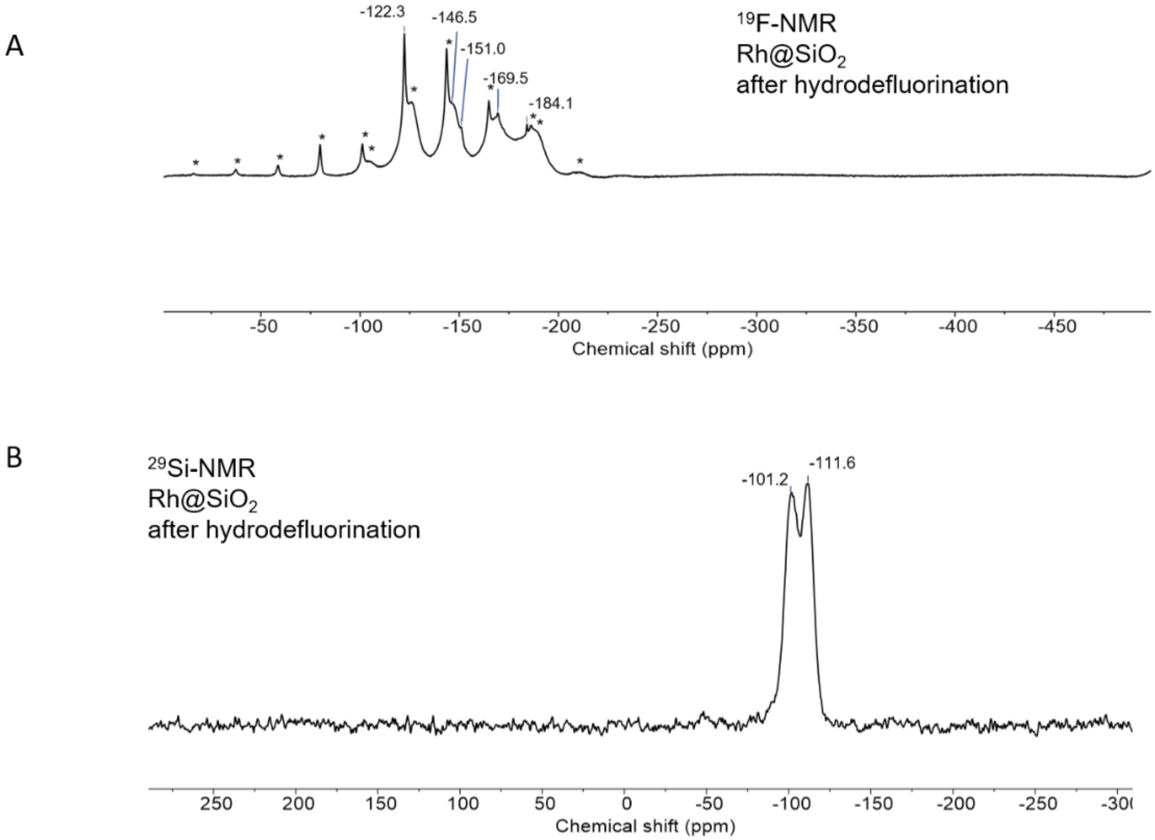

Figure S14: CP-MAS Solid state NMR of Rh@SiO_2 (A: ^{19}F and B: ^{29}Si), after hydrodefluorination of 4-fluoroacetophenone (see Table S3, Entry 1). *rotational sidebands.

Table S1: Recycling Test for Rh@SILP(Ph_3-P-NTf_2) using acetophenone (**1**) as substrate.

Entry	Catalyst	Substrate	Product distribution [%]		
			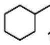 1b	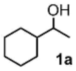 1a	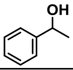
1	1 Run	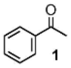 1	> 99	0	0
2	2 Run	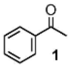 1	> 99	0	0
3	3 Run	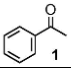 1	96	4	0
4	Supernatant from Run 3	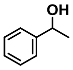	0[a]	0[a]	100

Reaction conditions: Rh@SILP(Ph_3-P-NTf_2) (20 mg, metal content: 0.002 mmol Rh), acetophenone (12.0 mg, 0.1 mmol, 50 eq.), n-heptane (375 mg), 100 °C, 18 h H_2 (50 bar), 500 rpm. Catalyst washed with 1 mL n-heptane between runs. [a] no conversion of phenylethanol to **1a** or **1b** observable/ no change in supernatant composition.

Table S2: Characterization data for silica, Rh@SILP(Ph_3-P-NTf_2) before and after catalysis including BET and elemental analysis.

Entry	Sample	IL loading [mmol/g SiO_2]	BET		Elemental analysis		
			Surface area [m^2/g]	Pore diameter [nm]	Rh [wt %]	P [wt %]	F [wt %]
1	Silica	/	325	7.8	/	/	/
2	SILP	/	292	7.5	/	/	/
3	Rh@SILP(Ph_3-P- NTf_2) (before catalysis)	0.4	271	8.7	0.89	1.14	4.2
4	Rh@SILP(Ph_3-P- NTf_2) (after catalysis, 1 run)	n.d	281	7.9	0.90	1.01	3.74
5	Rh@SILP(Ph_3-P- NTf_2) (after catalysis, 6 runs)	n.d	n.d	n.d	n.d	1.01	3.71

Reactions:

Table S3: Hydrogenation and hydrodeoxygenation of 4-fluoroacetophenone and acetophenone using Rh@SiO_2.

Entry	Catalyst	Substrate	Product distribution [%]		
			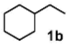 1b	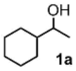 1a	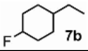 7b
1	Rh@SiO_2	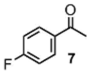 7	62	16	22
2	Rh@SiO_2	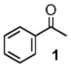 1	5	95	/
3	Rh@SiO_2 [a]	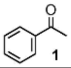 1	22	78	/

Reaction conditions: Rh@ SiO_2 (20 mg, metal content: 0.002 mmol Rh), acetophenone (12.0 mg, 0.1 mmol, 50 eq.), n-heptane (375 mg), 100 °C, 18 h H_2 (50 bar), 500 rpm. [a] After catalysis of Entry 1, catalyst washed with 1 mL n-heptane and 1 mL n-pentane.

Table S4: Influence of different substituents on the hydrodeoxygenation activity of Rh@SILP(Ph_3-P-NTf_2).

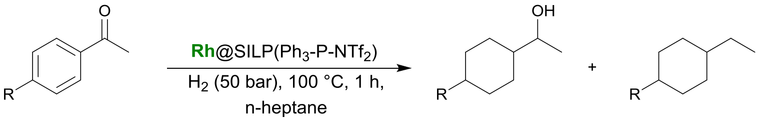

Entry	Substrate	Product Yield (%)[a]	
1	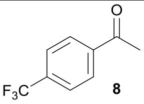 8	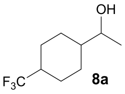 8a 50	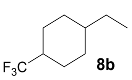 8b 50
2	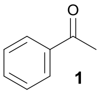 1	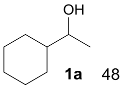 1a 48	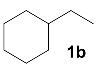 1b 52
3	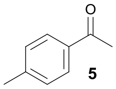 5	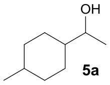 5a 42	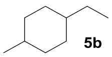 5b 58
4	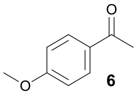 6	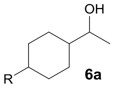 6a 29 [b]	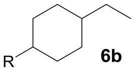 6b 71 [c]

Reaction conditions: Rh@SILP(Ph_3-P-NTf_2) (20 mg, metal content: 0.002 mmol Rh), substrate (0.1 mmol, 50 eq.), n-heptane (375 mg), 100 °C, H_2 (50 bar), 1 h, 500 rpm. [a] Distribution determined by GC-FID using tetradecane as an internal standard, conversion > 99%. [b] 1-(4-methoxycyclohexyl)ethan-1-ol (25%), cyclohexylethanol (4%). [c] 1-ethyl-4-methoxycyclohexane (40%), 1-ethyl-4-hydroxycyclohexane (22%), ethylcyclohexan (10%).

Table S5: Hydrogenation of benzylic substrates using Ru@SILP(Ph$_3$-P-NTf$_2$).

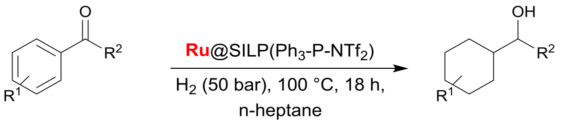

Entry	Substrate	Product Yield (%)[a]
1	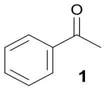 **1**	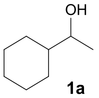 **1a** 89
2	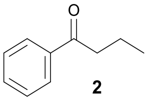 **2**	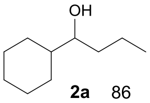 **2a** 86
3	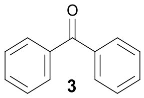 **3**	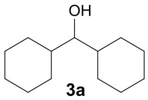 **3a** 86
4	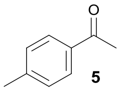 **5**	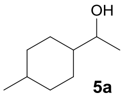 **5a** 90

Reaction conditions: Ru@SILP(Ph$_3$-P-NTf$_2$) (20 mg, metal content: 0.002 mmol Ru), substrate (0.1 mmol, 50 eq.), n-heptane (375 mg), 100 °C, H_2 (50 bar), 18 h, 500 rpm. [a] Distribution determined by GC-FID using tetradecane as an internal standard, conversion > 99 %. Remainders of reaction mixture: hydrodeoxygenated products.

Table S6: Hydrogenation of acetophenone and benzylideneacetone using Ru@SILP(Ph$_3$-P-NTf$_2$) at 175°C or 100 °C.

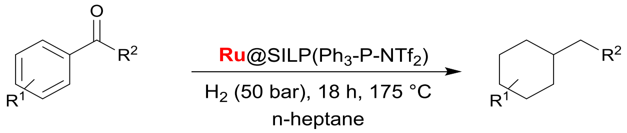

Entry	Substrate	Product Yield (%)[a]	Temp.
1	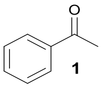 **1**	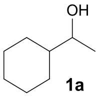 **1a** 92 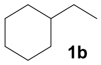 **1b** 8	175 °C
2	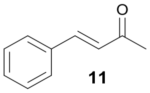 **11**	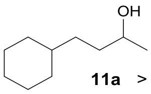 **11a** > 99	175 °C

3	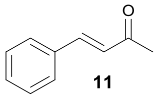 **11**	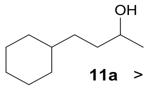 **11a** > 99	100 °C

Reaction conditions: Ru@SILP(Ph_3-P-NTf_2) (20 mg, metal content: 0.002 mmol Ru), substrate (0.1 mmol, 50 eq.), n-heptane (375 mg), H_2 (50 bar), 18 h, 500 rpm. [a] Distribution determined by GC-FID using tetradecane as an internal standard, conversion > 99.

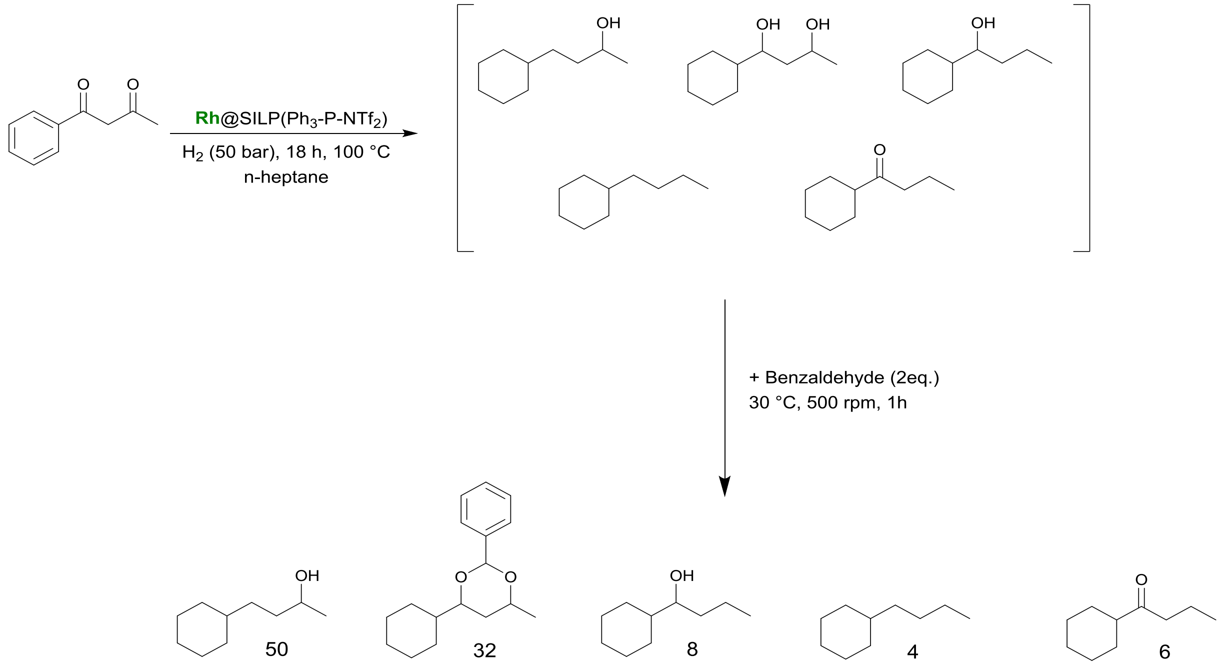

Scheme S1: Hydro(deoxy)genation of benzoylacetone at 100 °C. After reaction the autoclave was cooled down and depressurized, followed by the addition of benzaldehyde (2 eq. in regard to the substrate). Product distribution was determined by GC-FID using tetradecane as an internal standard.

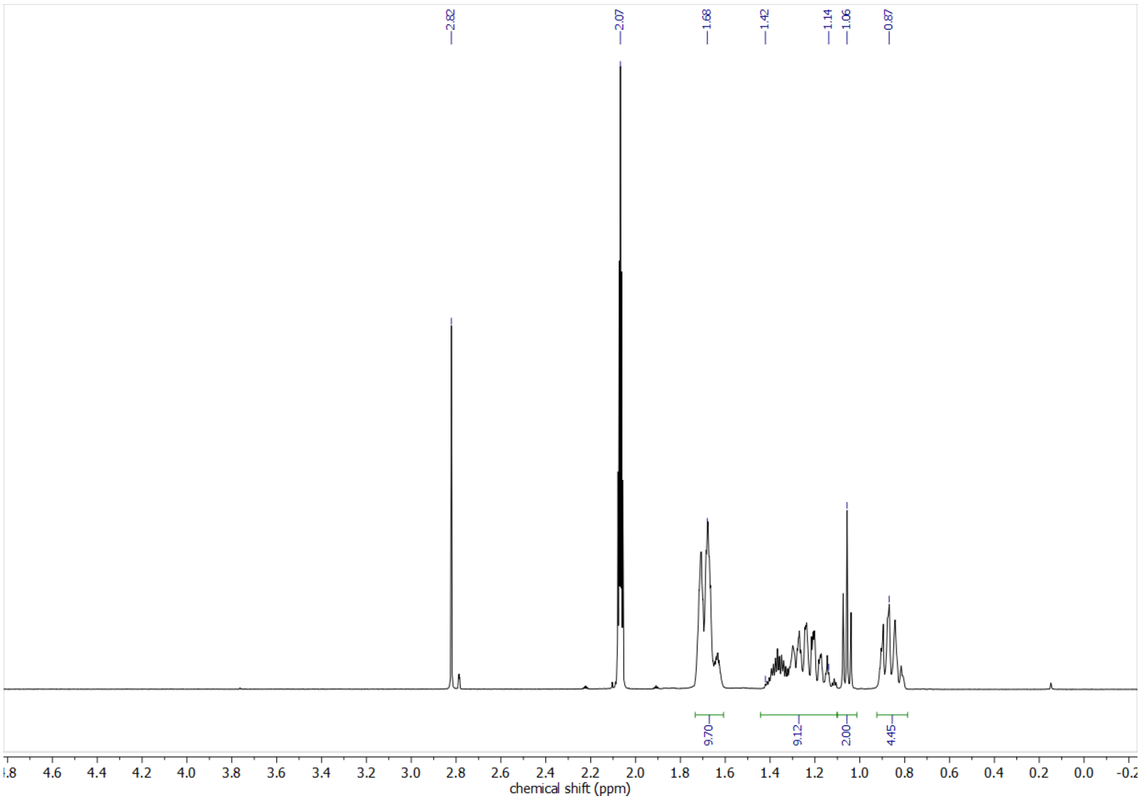

Figure S15: ^{1}H-NMR of hydrodeoxygenation of benzophenone **3** after isolation of the product **3b** (dicyclohexylmethane). *^{1}H NMR (400 MHz, $(CD3)_2CO$, δ): 0.80-0.92 (m, 4H), 1.06 (t, 2H), 1.10-1.42 (m, 9H), 1.61-1.74 (m, 9H).*

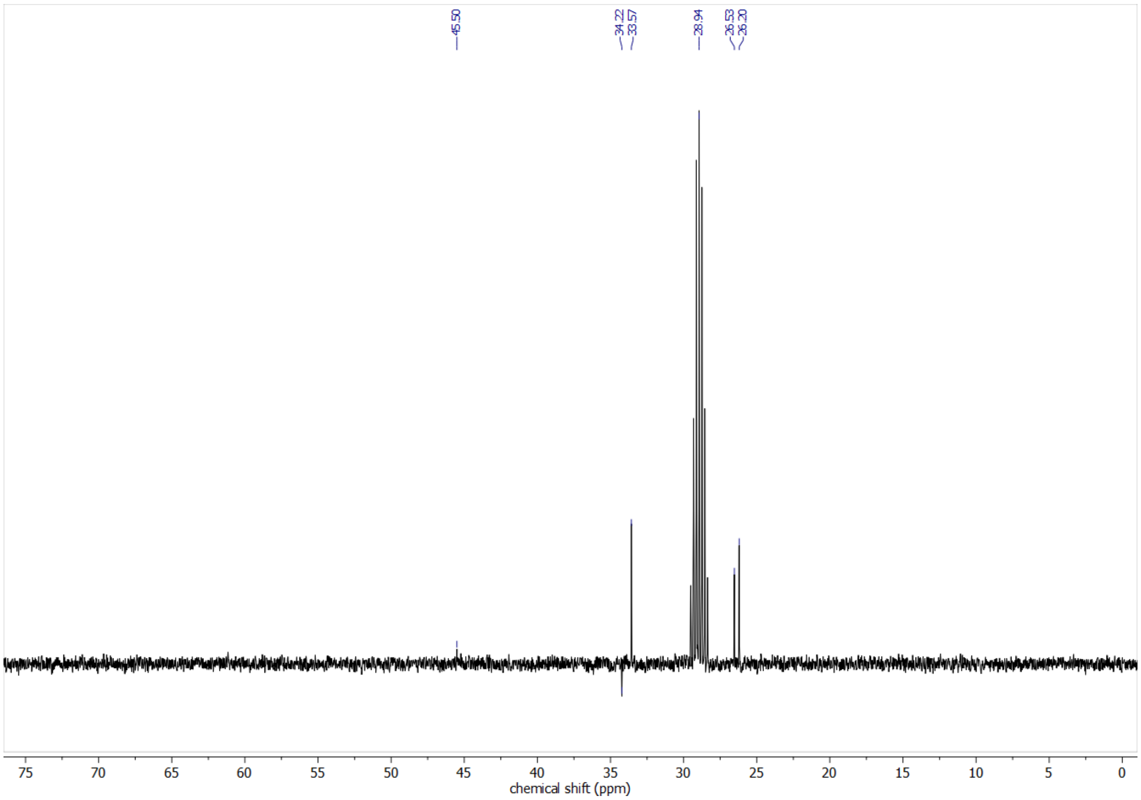

Figure S16: ^{13}C-NMR of hydrodeoxygenation of benzophenone **3** after isolation of the product **3b** (dicyclohexylmethane). *^{13}C NMR (100 MHz, $(CD3)_2CO$, δ): 26.2 (s, 4C), 26.5 (s, 2C), 33.5 (s, 2C), 34.2 (s, 4C), 45.5 (s, 1C).*

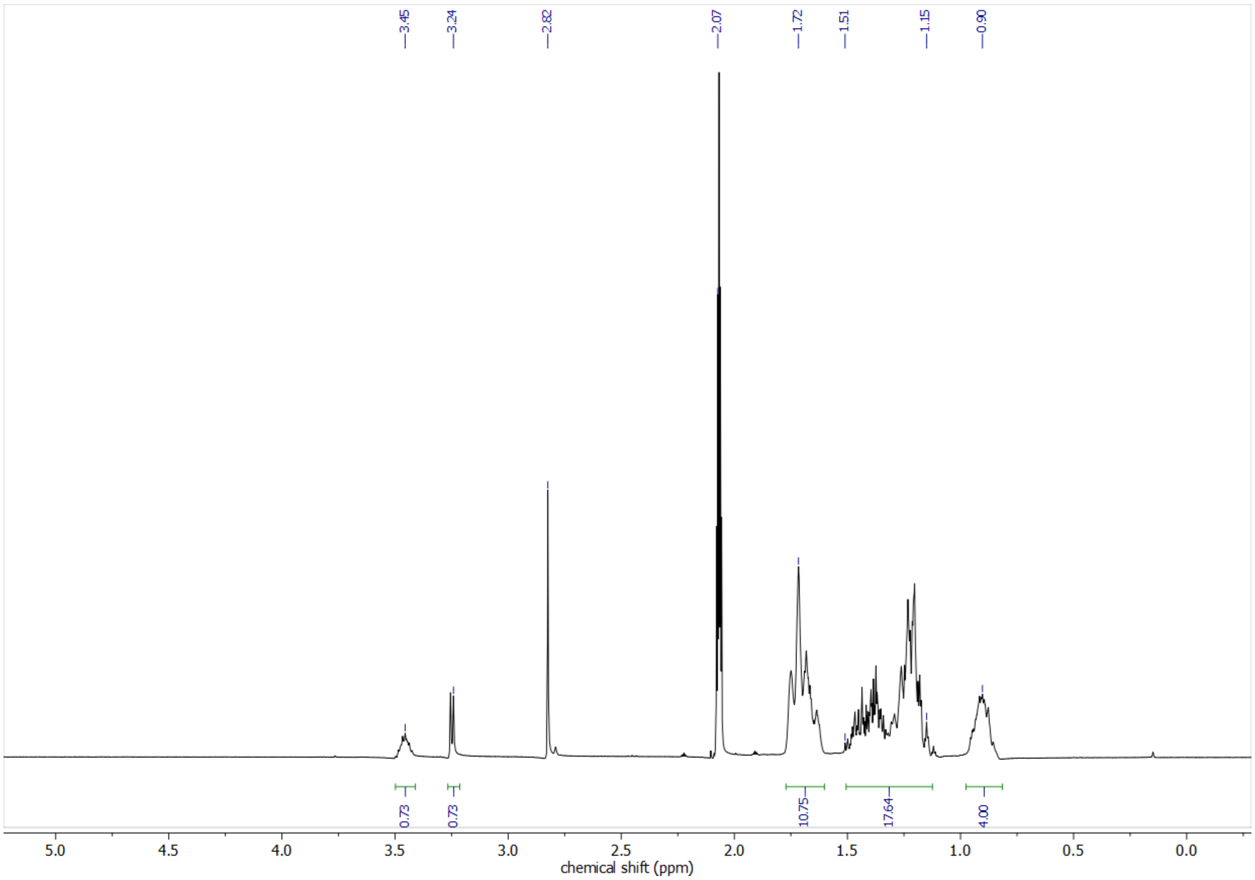

Figure S17: ^{1}H-NMR of hydrodeoxygenation of trans,trans-dibenzylideneacetone **16** after isolation of the product **16a** (1,5-dicyclohexylpentan-3-ol). *^{1}H NMR (400 MHz, (CD3)2CO, δ): 0.83-0.96 (m, 4H), 1.13-1.52 (m, 16H), 1.60-1.78 (m, 10H), 3.25 (d, J=5.2 Hz, 1H), 3.42-3.50 (m,1H).*

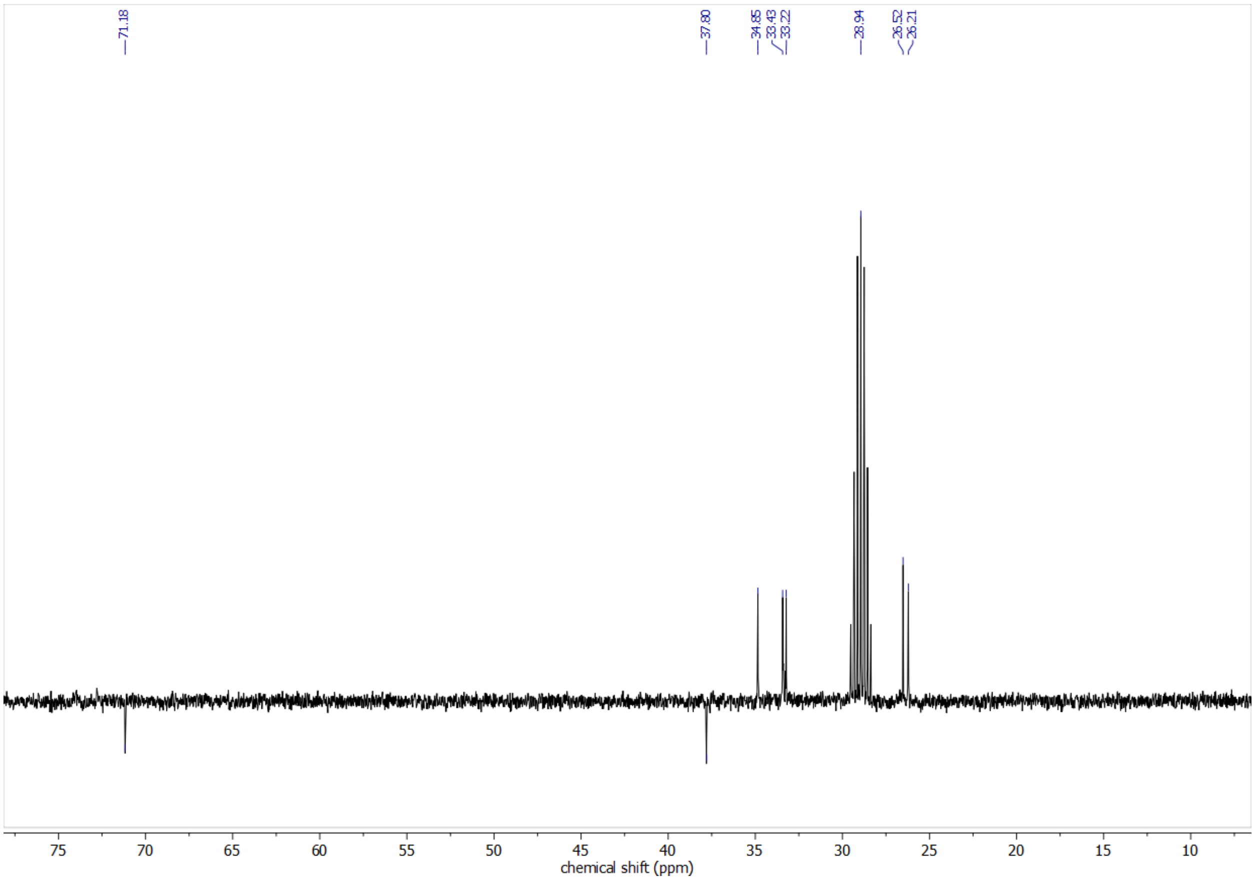

Figure S18: ^{13}C-NMR of hydrodeoxygenation of trans,trans-dibenzylideneacetone **16** after isolation of the product **16a** (1,5-dicyclohexylpentan-3-ol). *^{13}C NMR (100 MHz, $(CD3)_2CO$, δ): 26.2 (s, 4C), 26.5 (s, 2C), 33.2 (s, 2C), 33.4 (s, 2C), 34.8 (s, 2C), 37.8 (s, 4C), 71.2 (s, 1C).*

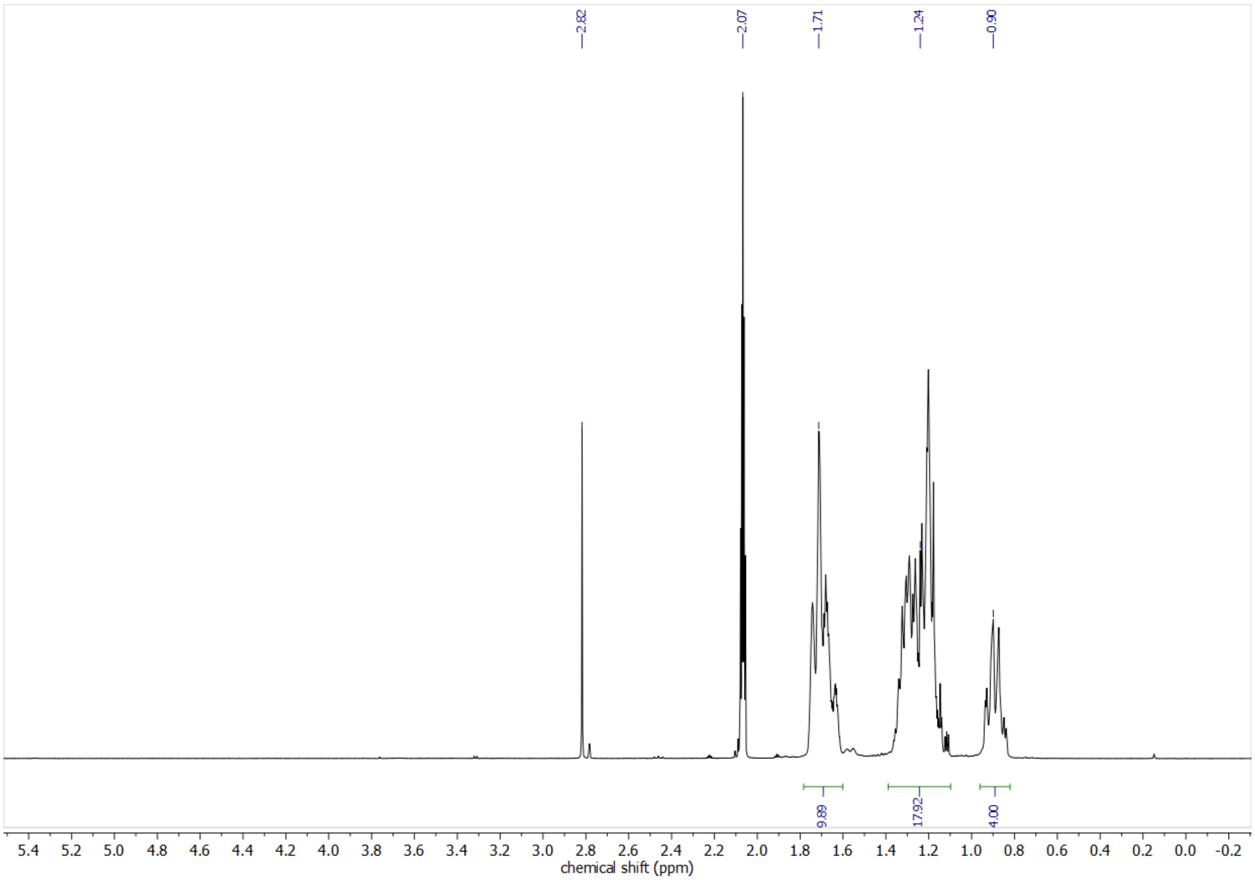

Figure S19: ^{1}H-NMR of hydrodeoxygenation of trans,trans-dibenzylideneacetone **16** after isolation of the product **16b** (1,5-dicyclohexylpentane). *^{1}H NMR (400 MHz, (CD3)$_2$CO, δ): 0.83-0.96 (m, 4H), 1.13-1.38 (m, 18H), 1.60-1.77 (m, 10H).*

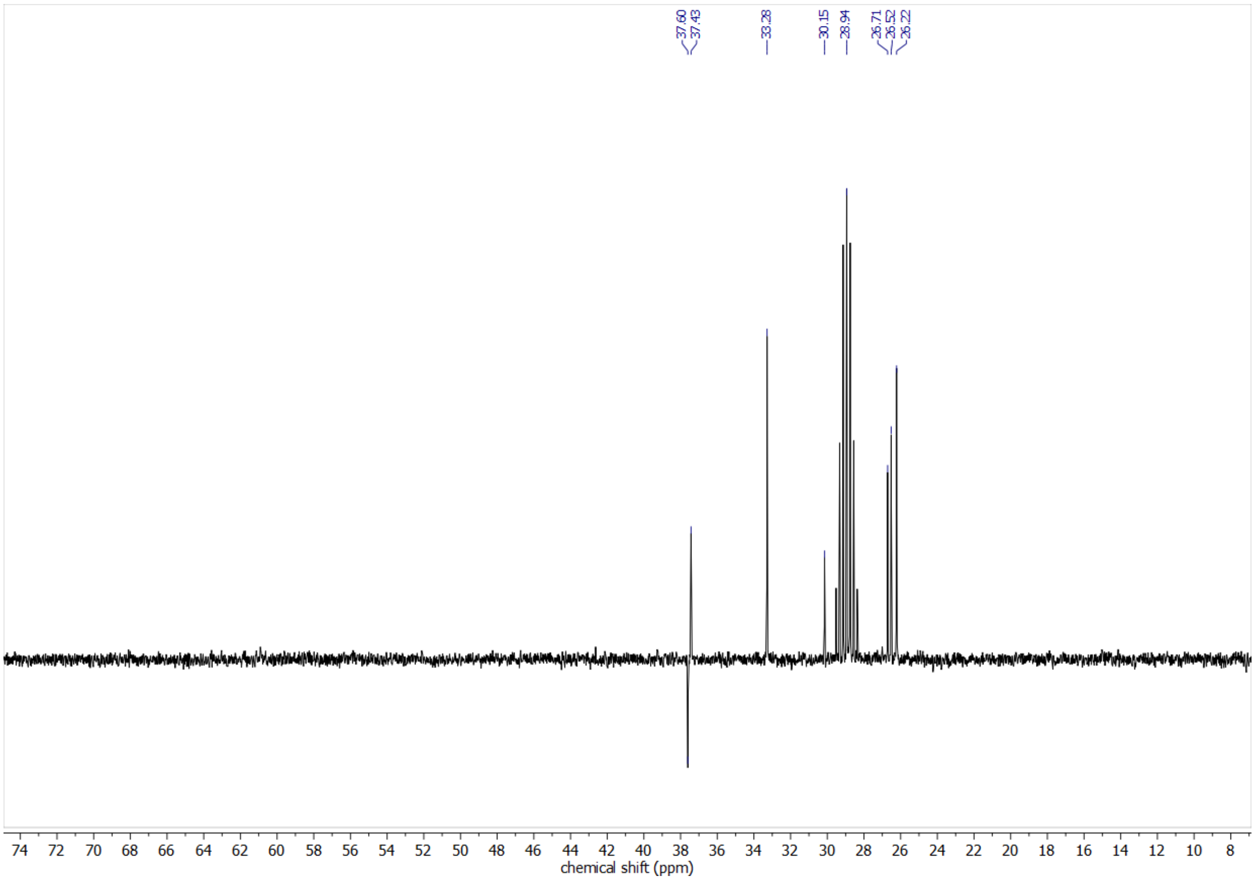

Figure S20: ^{13}C-NMR of hydrodeoxygenation of trans,trans-dibenzylideneacetone **16** after isolation of the product **16b** (1,5-dicyclohexylpentane). *^{13}C NMR (100 MHz, $(CD3)_2CO$, δ): 26.2 (s, 4C), 26.5 (s, 2C), 26.7 (s, 2C), 30.1 (s, 1C), 33.3 (s, 4C), 37.4 (s, 2C), 37.6 (s, 2C).*

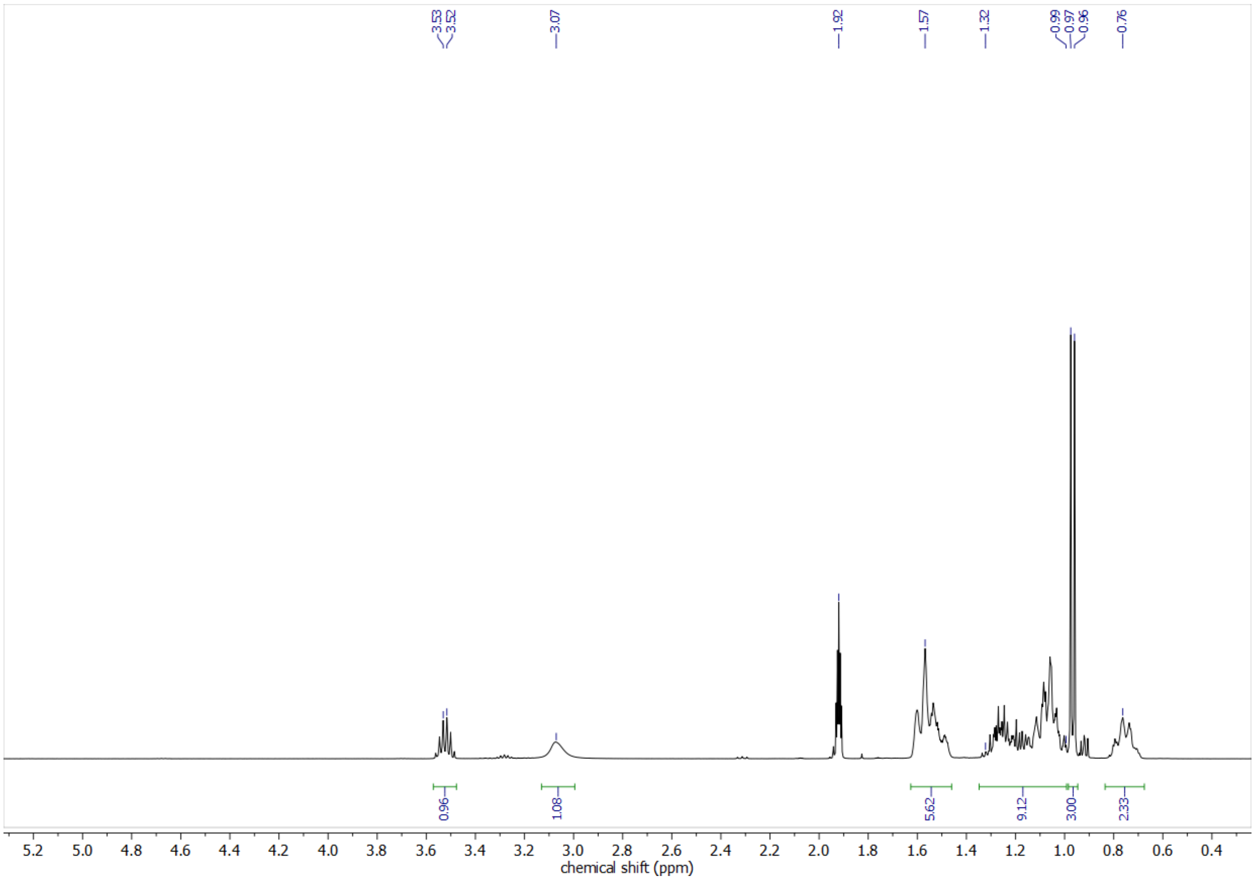

Figure S21: ^{1}H-NMR of hydrodeoxygenation of benzylideneacetone **11** after isolation of the product **11a** (4-cyclohexylbutan-2-ol). *^{1}H NMR (400 MHz, (CD3)$_2$CO, δ): 0.69-0.82 (m, 2H), 0.97 (d, J=6.2Hz, 3H), 1.01-1.34 (m, 8H), 1.46-1.62 (m, 5H), 3.07 (s, 1H), 3.52 (h, J=6.0 Hz 1H).*

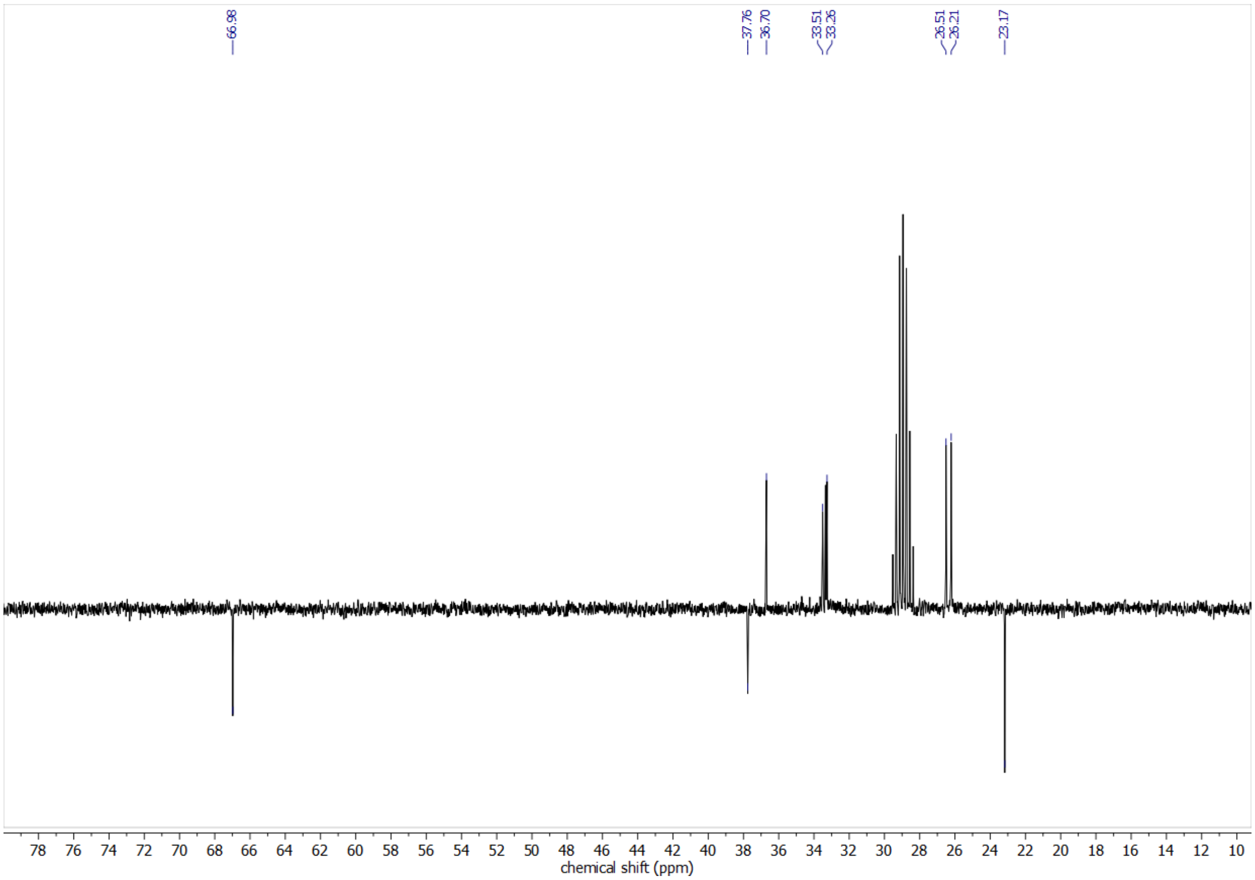

Figure 22: ^{1}H-NMR of hydrodeoxygenation of benzylideneacetone **11** after isolation of the product **11a** (1,5-4-cyclohexylbutan-2-ol). *^{13}C NMR (100 MHz, $(CD3)_2CO$, δ): 23.2 (s, 1C), 26.2 (s, 2C), 26.5 (s, 1C), 33.2 (s, 1C), 33.5 (s, 1C), 36.7 (s, 2C), 37.8 (s, 1C) 67.0 (s, 1C).*

References

[1] John, K. D., Eglin, J. L., Salazar, K. V., Baker, R. T., Sattelberger, A. P., Serra, D. and White, L. M. (2014). Tris(Allyl)Iridium and -Rhodium. In Inorganic Syntheses: Volume 36 (eds G. S. Girolami and A. P. Sattelberger). doi:10.1002/9781118744994.ch32

[2] K. L. Luska, P. Migowski, S. El Sayed, W. Leitner, *Angew. Chem., Int. Ed.* **2015**, *54*, 15750-15755.

[3] S. R. Ghanta, M. H. Rao, K. Muralidharan, *Dalton Trans* **2013**, *42*, 8420-8425.
